# Putative α7-selective ligands interact with α9-containing nicotinic acetylcholine receptors and modulate immune functions of human mononuclear phagocytes

**DOI:** 10.3389/fimmu.2026.1773637

**Published:** 2026-03-11

**Authors:** Mona Mobasher, Arik J. Hone, Malin Pilzecker, Daria Bozic, Andreas Hecker, J. Michael McIntosh, Karl N. Kirschner, Veronika Grau, Roger L. Papke, Hina Andleeb, Katrin Richter

**Affiliations:** 1 Institute for Functional Gene Analytics (IFGA), Bonn-Rhein-Sieg University of Applied Sciences, Rheinbach, Germany; 2 Laboratory of Experimental Surgery, Department of General and Thoracic Surgery, Justus Liebig University Giessen, Giessen, Germany; 3 School of Biological Sciences, University of Utah, Salt Lake City, UT, United States; 4 Department of Psychiatry, University of Utah, Salt Lake City, UT, United States; 5 George E. Whalen Veterans Affairs Medical Center, Salt Lake City, UT, United States; 6 Department of Computer Science and Institute of Technology, Resource and Energy-Efficient Engineering (TREE), Bonn-Rhein-Sieg University of Applied Sciences, Sankt Augustin, Germany; 7 Department of Pharmacology and Therapeutics University of Florida, Gainesville, FL, United States; 8 School of Pharmacy, Concordia University Wisconsin, Mequon, WI, United States

**Keywords:** cholinergic anti-inflammatory system, CHRNA10, CHRNA7, CHRNA9, docking, interleukin-1β, mononuclear phagocytes, nicotinic acetylcholine receptors

## Abstract

**Introduction:**

Nicotinic acetylcholine receptors (nAChRs) on immune cells are promising therapeutic targets for the treatment of inflammatory diseases and pain. Both α7 and α9* nAChRs (*denotes the potential presence of other nAChR subunits) have been implicated as mediators of the cholinergic anti-inflammatory system (CAS). This study investigated the binding sites of α7-selective ligands on these receptors and their effects on ATP-dependent release of the pro-inflammatory cytokines interleukin (IL)-1β and IL-18 by human mononuclear phagocytes.

**Materials and Methods:**

The effects of classical ligands (e.g. ACh, nicotine), unconventional (phosphocholine), putative α7-specific ligands (S24795, PNU-282987 and methyllycaconitine), on the ATP-induced IL-1β release were studied in lipopolysaccharide-primed human monocytic THP-1 cells and THP-1-derived macrophages. Electrophysiological two-electrode voltage-clamp measurements were conducted on *Xenopus laevis* oocytes expressing human α7, α9 or α9α10 nAChRs. Molecular docking was performed using the crystal structure of the homomeric human α7 receptor (PDB ID: 7EKI) and a modeled pentameric assembly of the homomeric α9 extracellular domain (PDB ID: 6HY7). In addition, the homomeric α10 extracellular domain was generated by homology modeling using 6HY7 as the template.

**Results:**

In cytokine-release experiments, the nAChR agonists efficiently inhibited ATP-mediated IL-1β release. This inhibitory effect was reversed by specific antagonistic conopeptides [V11L;V16D]ArIB (α7 antagonist) and RgIA4 (α9 and α9α10 antagonist), indicating the involvement of nAChRs containing subunits α7, α9 and/or α10. Electrophysiological measurements suggested an interaction of putative α7-specific ligands with human α9* nAChRs. In molecular docking simulations, all tested ligands showed reasonable binding affinity to homomeric α7, α9 and α10 nAChR models near the C-loop region of the binding pocket.

**Conclusion:**

Our findings provide a more nuanced framework for interpreting the roles of nAChR subtypes in non-neuronal immune modulation, highlighting the complexity and potential importance of α9* nAChRs in the context of inflammation and innate immunity. The results underscore the importance of considering the nAChR subunit α9 when developing α7-selective ligands for immunomodulation and provides novel insights into the role of α9* nAChRs as potential therapeutic targets for inflammatory diseases and pain.

## 1 Introduction

Nicotinic acetylcholine receptors (nAChRs) are known historically as ligand-gated ion channels, which play an indispensable role in neurotransmission including the motor endplate (1, 2). A functional nAChR consists of a pentameric complex of subunits that can assemble as homomers and heteromers (3). Each subunit consists of an extracellular domain (ECD), four transmembrane domains (TMD) interconnected with linking loops and one variable cytoplasmic domain (3–5). The ECD houses the site where orthosteric ligands bind and connects to the TMDs, which form the ion channel pore (3–5). In mammals 16 different nAChR subunits have been identified. The nAChR subunits are divided into type α (α1 – α7, α9, α10) and non-type α (β1 – β4, γ, δ, or ϵ) (2, 3, 6). The orthosteric ligand binding-site is located between two adjacent subunits (7). According to their typical cellular expression patterns, nAChRs can be divided into muscular or neuronal types. Muscular nAChRs are formed in adult muscle from two α1 and one β1, δ and ϵ subunits each, and are found at neuromuscular endplates (8). During embryogenesis, a γ subunit is expressed instead of the ϵ subunit (8). Neuronal nAChRs occur both as homopentamers of the α7 or α9 subunits, heteropentamers composed of the subunits α7 and β2 or α9 and α10, as well as in numerous other combinations of two or three α subunits (α2 – α4 or α6) with two or three β subunits (β2 or β4) (9).

A new chapter in cholinergic research was written with the discovery of the “non-neuronal cholinergic system”. Studies have shown that the essential components and receptors of the cholinergic system - such as choline acetyltransferase, ACh esterase, ACh, muscarinic AChRs and nAChRs - are also expressed by non-neuronal cells in mammals (1, 10, 11). Most immune cells such as T and B cells, monocytes, macrophages and dendritic cells, express the essential components of the cholinergic system for the synthesis, transport and release of ACh, as well as various nAChR subunits (e.g., α2, α5, α6, α7, α9, α10 and β2) (12–14). The discovery that immune cells have nAChRs, which can be stimulated to modulate and most commonly reduce the release of pro-inflammatory cytokines (15, 16), has sparked interest in cholinergic anti-inflammatory systems (CAS). Consequently, various therapeutic approaches for the treatment of inflammatory diseases and neuropathic pain have emerged (16–18). For example, activation of nAChRs by classical ligands (e.g. ACh, nicotine) has a protective effect on various inflammatory diseases, such as ulcerative colitis, rheumatoid arthritis and gout (16, 19, 20). For nAChRs with the α7 subunit, several studies have shown that its activation leads to a mitigation of cytokine release and can inhibit the expression of pro-inflammatory cytokines at the translational level (13–15, 19, 21–23).

The canonical mode of nAChRs is ionotropic (2, 3). Numerous downstream signaling mechanisms are activated in response to nAChR stimulation of immune cells, though they are only partially understood (for review see (24)). A completely different mode of action was first shown for α7 nAChRs. In studies on α7 nAChRs, the CAS activity is associated with receptor activation and, to a larger degree, with receptor desensitization (25–29). Indeed, nAChR “silent agonists”, substances that induce metabotropic but not ionotropic functions at α7 nAChRs, seem to be among the most effective modulators of CAS (27). In microglial cells, it was shown that the α7 nAChR silent agonist NS6740 inhibited lipopolysaccharide (LPS)-induced release of tumor necrosis factor (TNF)-α more effectively than the partial α7 agonist GTS-21 (25). Despite the higher concentrations needed for NS6740 these data indicate that functional silence (NS6740) seems to be more effective at down modulating inflammatory signaling than weak activation of α7 nAChRs by GTS-21 (25). Thus, nAChR silent agonists represent a promising therapeutic approach for the treatment of inflammatory diseases (27).

The discovery of the dupα7 protein (gene *CHRFAM7A*) has added an additional layer of regulatory complexity to the cholinergic control of immune functions. *CHRFAM7A* is a human-specific duplication of the *CHRNA7* gene, and its overexpression is linked to neurocognitive diseases (schizophrenia, bipolar disorder) and immune disorders (16, 30–33). Moreover, in recent years it has been shown that in addition to α7, other nAChR subunits, such as α9 and β2, can also induce anti-inflammatory effects (12, 34–38).

nAChRs containing α9* (*denotes homomeric or the potential presence of other nAChR subunits) subunits have also been identified as effective mediators of CAS (20, 34, 37, 39, 40). In previous studies, we identified a cholinergic control mechanism of the ATP-induced release of interleukin (IL)-1β (12, 39, 41–43). The inflammatory mediator IL-1β is a highly potent pro-inflammatory cytokine of the innate immune system that is mainly secreted by mononuclear phagocytes (44–46). We provided evidence that activation of nAChRs containing subunits α9, α7 and/or α10 (here denoted as α9* nAChRs) inhibits the ATP-sensitive P2X7 receptor (P2RX7), and thus the NLRP3 (NLR family, pyrin domain containing protein 3) inflammasome-dependent release of IL-1β (12, 41, 42). As summarized in **Table 1**, cholinergic control is mediated by classical agonists like ACh and choline. Moreover, we identified phosphocholine (PC)-bearing molecules that function as novel, unconventional ligands of α9* nAChRs that efficiently inhibited ATP-mediated IL-1β release by mononuclear phagocytes with silent agonist-like functions (12, 41, 42) (**Table 1**). In mononuclear phagocytes, it is generally questioned whether CAS activity is associated with ion channel activation or rather with metabotropic nAChR functions (for review see (24)). Moreover, the structure of these unconventional nAChRs is unknown. The interaction of nAChR subunits α7, and/or α9/α10 was first described in isolated rat dorsal root ganglion neurons (47), and later in a mast cell line (48). In addition, we demonstrated that canonical nAChR agonists as well as PC inhibit ATP-induced release of IL-1β by mononuclear phagocytes via nAChR subunits α7, α9 and α10 whereas more complex metabolites of phosphatidylcholines elicited the inhibitory mechanism in human monocytes via interaction of the two nAChR subunits α9 and α10 (12, 41, 42) (**Table 1**).

**Table 1:** Classical, unconventional and synthetic ligands of nAChRs, their effect on the ATP-induced release of IL-1β by mononuclear phagocytes, as well as on the ionotropic function of heterologously expressed human nAChR subunits (i.e., homomeric α7 and α9; heteromeric α9/α10).

Ligand	Effect on ATP-induced IL-1β release by mononuclear phagocytes	Effect on heterologously expressed nAChRs	Effect on ACh- or choline-induced ion currents
ACh	Inhibition via subunits α7, α9, α10 (41, 42).	Agonist of α7, α9 and α9α10 (42).	n.a.
Choline	Inhibition via subunits α7, α9, α10 (41, 42).	Agonist of α7, α9 and α9α10 (41).	n.a.
Nicotine	Inhibition via subunits α7, α9, α10 (42).	α7 agonist; α9α10 antagonist (40).	Reversibly inhibits ACh-induced ion currents of α9α10 nAChRs with an IC_50_ of 437 ± 73 μM (40).
PC	Inhibition via subunits α7, α9, α10 (12, 39, 41, 42, 75).	No ionotropic effects on α7, α7α9, α7α9α10, α9α10 nAChRs (39, 41, 42).	Reversibly inhibits choline-induced ion currents of α9α10 nAChRs (41). Effects on ACh-induced ion currents of α9α10 significantly lower and no effect on α7 nAChRs (39).
*p*CF3-diEPP	Inhibition via subunits α7, α9* (39).	Silent agonist for α7 nAChRs. No ionotropic effects on α9- and α9α10-nAChRs (39).	Reversibly inhibits ACh-induced ion currents of α7, α9 and α9α10 nAChRs with 30 μM. At 1 μM, no effect on α7 nAChRs (39).
*p*CN-diEPP	Inhibition via subunits α7, α9*. In addition, antagonizing the effect of ACh and PC (40).	Silent agonist for α7 and agonist for α9α10 nAChRs (40).	n.a.
*m*CN-diEPP	None, but antagonizes the effect of ACh, PC, and *p*CN-diEPP (40).	Partial α7 antagonist, as well as antagonist of α9α10 nAChRs (40).	Reversibly inhibits ACh-induced ion currents of α7 and α9α10 nAChRs (40).
MLA	Not tested.	Antagonist of α7 nAChRs (40).	Reversibly inhibits ACh-induced ion currents of α7, α9 and α9α10 nAChRs (40).

ACh, acetylcholine; G-PC, glycerophosphocholine; IL, interleukin; LPC, lysophosphatidylcholine; MLA, methyllycaconitine; *m*CN-diEPP, 4-(3-cyanophenyl)-1,1-diethylpiperazine-1-ium; n.a., not applicable; nAChRs, nicotinic ACh receptors; *p*CF3-diEPP, *p*CF3-N,N-diethyl-N´-phenyl-piperazine; PC, phosphocholine; *p*CN-diEPP, 4-(4-cyanophenyl)-1,1-diethylpiperazine-1-ium.

As research groups discover new ligands that modulate specific nAChR subunits, there is a tendency to classify them as α7-specific (49–52) or α9*-specific (53–57). Numerous ligands that selectively activate, desensitize, antagonize, or allosterically modulate the α7 nAChR have been identified (52). However, ligand binding and their subsequent modulation can be diverse due to the complexity of nAChR compositions, their distribution across different tissues, and modes of action. Research on α9* nAChRs is currently challenging due to the limited availability of selective ligands for α9 and the absence of specific antibodies against α7 and α9 (24, 58, 59). In addition, α9 subunits are known to combine with α10 subunits to form α9α10 nAChRs with kinetic properties slightly different from homomeric α9 nAChRs (40, 60, 61). The α10 subunit has long been thought to require α9 subunits for functional expression (62). However, evidence for functional homomeric α10 nAChRs was provided by exposure of α10 nAChR-expressing oocytes to certain alkaloids, for example methyllycaconitine (MLA) or strychnine (63) and human α10 subunits have now been shown to self-assemble (64). We recently tried to mimic the simultaneous occurrence of human nAChR subunits α7, α9 and α10 in the heterologous expression system of *X. laevis* oocytes and identified separate receptor populations including a potential population of homomeric α10 nAChRs (40) (**Table 1**). Although MLA is frequently described as an α7-selective antagonist that has been used in other studies to implicate an α7-dependent mechanism in CAS activity (28, 65, 66), it has been argued that it behaves more like an inverse agonist than a competitive antagonist (67, 68). Its selectivity remains, however, a subject of discussion. In line with previous findings (69), our study provides further evidence that MLA is not exclusively selective for α7 nAChRs but also exhibits sensitivity towards α9* nAChRs (40) (**Table 1**).

**Figure 1:**
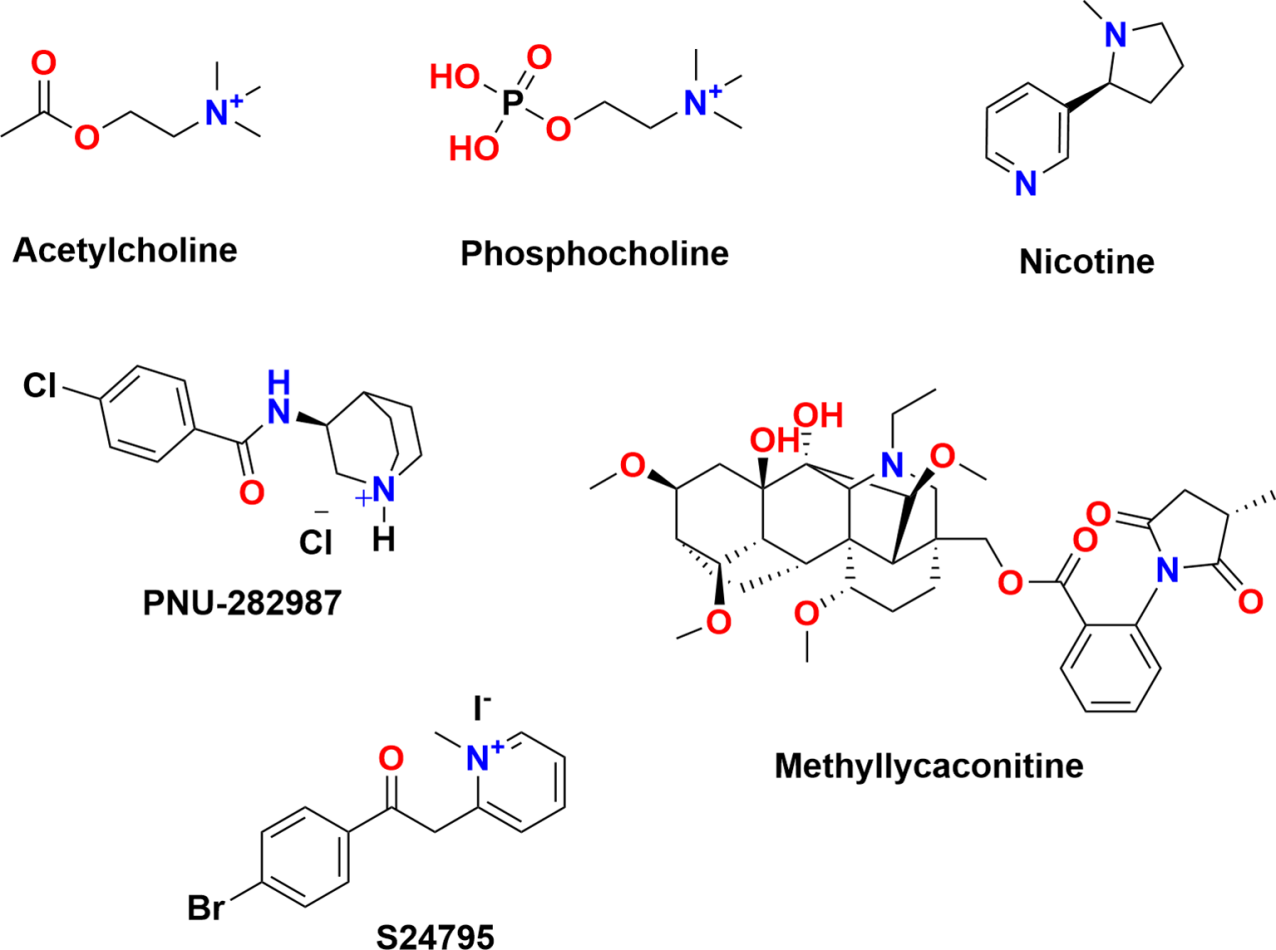
Chemical structures of the molecules investigated to modulate the ATP-induced release of the pro-inflammatory cytokine interleukin (IL)-1β by mononuclear phagocytes.

This prompted us to investigate the ability of the putative α7-specific ligands S24795 (70, 71), PNU-282987 (72–74), and MLA (**Figure 1**) to modulate ATP-induced IL-1β release by mononuclear phagocytes. We provide evidence that these putative α7-specific agonists interact with unconventional α9* nAChRs to shape the immune function of mononuclear phagocytes. Specifically, electrophysiological two-electrode voltage-clamp (TEVC) measurements on overexpressed human α9* nAChRs show modulation by putative α7-selective agonists. To gain atomistic insight, we include an *in silico* docking study on homomeric human α7, α9 and α10 nAChRs of classical (ACh, nicotine), putative α7-selective agonists (S24795, PNU-282987) and MLA, that support the experimental activity data.

## 2 Materials and methods

### 2.1 Chemicals and reagents

ACh chloride (Cat# A6625), apyrase (Cat# A6410), choline chloride (Cat# C7017) dimethyl sulfoxide (DMSO, Cat# D2650), LPS (*E. coli* O111:B4, Cat# L2630; *E. coli* O26:B6, Cat# L2654), methyllycaconitine citrate salt (MLA; Cat# M168), nigericin sodium salt (Cat# N7143), phorbol 12-myristate 13-acetate (PMA, Cat# P1585), and PC chloride calcium salt tetrahydrate (Cat# P0378) were purchased from Merck (Darmstadt, Germany). All chemicals for saline Ringer’s bufier preparations were purchased from Sigma (Merck, St. Louis, MO, USA). Gibco penicillin-streptomycin solution and L-glutamine solution were purchased from Thermo Fisher Scientific (Dreieich, Germany). BzATP [2’(3’)-O-(4-benzoyl-benzoyl)ATP trieethylammonium salt] was purchased from Jena Bioscience (Jena, Germany), S24795 (2-[2-(4-bromophenyl)-2-oxoethyl]-1-methylpyridinium iodide, Cat# 3518) and PNU-282987 (N-(3R)-1-azabicyclo[2.2.2]oct-3-yl-4-chlorobenzamide, Cat# 2303) from Tocris (Bio-Techne, Wiesbaden-Nordenstadt, Germany) and recombinant human interferon (INF)-γ from R&D Systems (Minneapolis, MN, United States; Cat# 285-IF-100). PMA, S24795 and PNU-282987 were dissolved in DMSO. Nigericin was dissolved in ethanol. When appropriate, control experiments were performed with the corresponding concentrations of DMSO/ethanol without drugs. The conopeptides [V11L,V16D]ArIB (specific antagonist of α7) (76–78) and RgIA4 (antagonizing nAChRs composed of subunits α9 and α10 (79–81) were produced and characterized as described previously (80, 82).

The concentrations of nAChR ligands used in this study were selected based on previously published pharmacological studies demonstrating functional modulation of nAChR subtypes in the *Xenopus laevis* oocyte overexpression system and immune signaling pathways (see **Table 1**). Because detailed affinity and efficacy data for α9* nAChRs in immune cells are limited, we employed concentration ranges commonly used to probe functional activity and immunomodulating mechanisms, while confirming the absence of cytotoxic effects by measuring LDH activity.

### 2.2 THP-1 cells

THP-1 cells (German Collection of Microorganisms and Cell Cultures, Cat# ACC16) were cultured under 5% CO_2_ atmosphere at 37 °C using RPMI 1640 medium from Sigma (Merck, Cat# R8758) or Capricorn (Ebsdorfergrund, Germany, Cat# RPMI-A) supplemented with 10% fetal bovine serum (FBS) from Capricorn (Cat# FBS-16A). For IL-1β release experiments, monocytic THP-1 cells were resuspended in FBS-free RPMI medium and 0.5 × 10^6^ cells/0.5 ml/well were seeded into 48-well plates (Greiner Bio-One, Frickenhausen, Germany). Cells were left untreated or primed with 1 µg/ml LPS (*E. coli* O26:B6) (39, 40, 83). After priming, BzATP (100 µM) or nigericin (50 µM) was added for 40 min in the presence or absence of cholinergic agonists and antagonists. Nigericin is a bacterial toxin that forms pores in the plasma membrane and induces inflammasome assembly independent of ATP (84, 85). As ATP might pass through the pores formed by nigericin, in these experiments 0.5 U/ml apyrase was added together with nigericin to ensure that the nigericin-induced release of IL-1β is ATP-independent. At the end of the experiments, cells were spun down (500 g, 8 min, 4 °C) and the cell-free supernatants were collected and stored at −20 °C for later cytokine measurements. Moreover, lactate dehydrogenase (LDH) activity was determined to estimate cell death.

For generating THP-1 cell-derived M1-like macrophages, 6 x 10^6^ cells were seeded in T75 cell culture flasks (Sarstedt, Nümbrecht, Germany) and incubated with 50 nM PMA in complete medium (10% FBS) for 24 h, followed by 24 h resting in fresh complete medium without PMA. Thereafter, cells were cultured in fresh complete medium supplemented with 10 ng/ml human IFN-γ and 10 ng/ml LPS (*E. coli* O111:B4) for 48 h to induce cell difierentiation into M1-like macrophages as previously described (75, 83). On day 5 of differentiation, cells were washed once with Dulbecco´s phosphate buffered saline without calcium and magnesium (PBS; Merck Cat# D8537) and detached using TrypLE™ Express (Thermo Fisher Scientific, Cat# 12605010) according to the manufacturer’s protocol. Detached cells were then seeded in FBS-free RPMI 1640 medium in a density of 0.05 x 10^6^ cells in 96-well plates (Greiner). To investigate cytokine release, THP-1 cell-derived M1-like macrophages were handled as described for monocytic THP-1 cells.

### 2.3 Measurement of cytokines and LDH activity

Cytokine concentrations of IL-1β and IL-18 were measured in the above-described cell-free supernatants using the human IL-1 beta/IL-1F2 DuoSet enzyme-linked immunosorbent assay (ELISA) from R&D Systems (Cat# DY201) and human Total IL-18 DuoSet ELISA (Cat# DY318-05) according to the supplier’s instructions.

Bioactive IL-1α/β in the cell-free supernatants was quantified using the HEK-blue IL-1R1 reporter cell-line (InvivoGen, San Diego, CA, United States; Cat# hkb-il1r) via IL-1R-dependent activation of NF-κB/AP-1, resulting in the subsequent production of SEAP reporter expression. The assay was performed according to the supplier’s instructions. In brief, 20 µl of supernatants were added together with 5 x 10^4^ HEK-blue IL-1R1 cells per well in a flat-bottom 96-well plate (Greiner) and incubated overnight at 37 °C in 5% CO_2_. On the next day, 20 µl of these supernatants were transferred to a new flat-bottom 96-well plate (Greiner) and incubated with QUANTI-Blue (InvivoGen, Cat# rep-qbs) for 90 min at 37 °C in 5% CO_2_. The absorbance was measured at 635 nm on a CLARIOstar Plus plate reader (BMG LABTECH GmbH, Ortenberg, Germany). IL-1α/β-equivalent bioactive concentrations (pg/ml) were determined by interpolation from a 4-parameter logistic standard curve generated with a serial dilution of recombinant human IL-1β (InvivoGen, Cat# rcyc-hil1b) that was dissolved in FBS-free RPMI 1640 medium and run in parallel with the samples.

To measure LDH activity, the CytoTox 96^®^ Non-Radioactive Cytotoxicity Assay (Promega, Madison, WI, United States; Cat# G1780) was performed according to the manufacturer’s instructions. LDH activities determined in cell-free supernatants are given as percentage of 100% cell lysis control.

### 2.4 TEVC electrophysiology

Oocytes were harvested from mature female *Xenopus laevis* frogs (Papke lab, Nasco, Ft. Atkinson, WI; McIntosh lab, Xenopus 1, Dexter, MI). All procedures were approved by the respective Institutional Animal Care and Use Committees (U. Florida, approval 202002669; U. Utah, approval 17−07020). Frogs were anesthetized in 1.5 l frog tank water containing 1 g of MS-222 (3-aminobenzoate methanesulfonate; Millipore Sigma, St. Louis MO, United States; Cat# E10521) buffered with sodium bicarbonate (Millipore Sigma; Cat# R8758) (Papke lab) or with 0.4 wt/vol Tricaine−S (Thermo Fisher Scientific; Cat# 140520250; McIntosh lab) and sacrificed after removal of the ovarian lobes. Oocytes were isolated and stage V oocytes were used for cRNA injection.

Plasmid DNA encoding the human *CHRNA7* was obtained from J. Lindstrom (University of Pennsylvania, Philadelphia, PA, United States), human *CHRNA9* and *RAPSN* from K. Richter (40, 41), *CHRNA10* from J. M. McIntosh (41, 55, 86), and the human resistance-to-cholinesterase 3 (*RIC3*) clone from M. Treinin (Hebrew University, Jerusalem, Israel). The expression of nAChR subunits in *Xenopus laevis* oocytes is generally very low and unstable. Therefore, RNA for Rapsyn (87, 88) or RIC3 (89, 90) is co-injected to achieve stable nAChR expression without altering the pharmacological properties of nAChRs (89).

cRNAs were synthesized as described previously (39, 41, 55, 86) and injected as follows: Papke lab injected 4–6 ng α7 RNA plus 2–3 ng RIC3 RNA (2:1 ratio) in 50 nl water, or 12 ng α9 RNA and 3 ng Rapsyn RNA in 50 nl water. McIntosh lab injected 12 ng α9 RNA, 12 ng α10 RNA and 3 ng Rapsyn RNA (4:4:1 ratio per subunit) in 50 nl water. Oocytes were then incubated for 24–72 h before recording.

TEVC recordings were performed on an OpusXpress 6000A (Molecular Devices) (91) or a Warner Instruments OC−725C clamp (63, 86). Oocytes were perfused at ~2 ml min^-^¹ with a control solution (115 mM NaCl, 2.5 mM KCl, 1.8 mM CaCl_2_, 1 mg/ml BSA, 10 mM HEPES, pH 7.2). The membranes holding potential was set to –60 mV and oocytes were stimulated once every 60 s for a 1 s duration with 60 µM ACh. Once stable ACh-evoked currents were observed, the control solution was switched to one containing the compound of interest and the ACh-evoked responses monitored for changes in current amplitudes. After application of the compound, the perfusion solution was switched back to control solution. To probe antagonist effects of S24795 (30 µM), PNU−282987 (30 µM), and RgIA4 (200 nM) the perfusion buffer was swapped to include the test compound while maintaining the ACh stimulus. The current amplitudes were compared to control. For agonist assessment, after ACh application, the oocytes were stimulated with 30 µM PNU−282987 or S24795, and the resulting currents were benchmarked against the ACh−evoked responses. This protocol yielded quantitative measures of ligand potency and efficacy on α9α10 and α7−containing nAChRs expressed in the two experimental setups.

### 2.5 Statistical analyses and data processing

Data were analyzed using SPSS^®^ (Version 29, IBM^®^, Armonk, NY, USA). Multiple independent data sets were first analyzed by the non-parametric Kruskal–Wallis test. In case of a p ≤ 0.05, the non-parametric Mann–Whitney U test was performed to compare individual groups and again, a p ≤ 0.05 was considered as evidence for statistical significance. Dependent data sets were analyzed first by the Friedman test followed by the Wilcoxon signed-rank test. All numbers (n) of the individual experiments are indicated in the Results section and in the Figures. No outliers were excluded from the analyses. Due to inter-experimental variability in baseline cytokine concentrations, cytokine release was normalized to the corresponding control and expressed as relative change (%). The absolute cytokine concentrations are provided in the Results section and **Supplementary Figure S1**. The free and open-source software Inkscape Version 0.48.5 r10040 (licensed under the GPL) was used for visualization of the data.

### 2.6 Molecular docking - protein preparation

The crystal structure of the human homomeric α7 nAChR was retrieved from the Protein Data Bank (PDB ID: 7EKI). For residues with alternate conformations, the conformers with the highest occupancy values were retained. Carbohydrates and crystallographic water molecules were removed and missing heavy atoms were added using the PyMOL Molecular Graphics System (version 2.1 Schrödinger, LLC, https://pymol.org). Additionally, a homomeric pentameric ECD of the α9 nAChR was modeled based on its monomeric subunit structure (PDB ID: 6HY7). The pentameric assembly was generated in PyMOL by applying symmetry operations to reproduce the receptor’s physiological quaternary structure. To relax the model and eliminate steric clashes introduced during the assembly process, steepest descent energy minimization was performed using GROMACS. After the system was solvated with the TIP3P water model and neutralized with sodium ions (Na^+^). The CHARMM36 force field was applied throughout the system. In addition, the homomeric human α10 nAChR ECD was generated by homology modeling using MODELLER (Version 10.7, https://salilab.org/modeller/) (92). Homology modeling of the α10 nAChR ECD was based on the X-ray crystal structure of the human α9-ECD in complex with α-conopeptide RgIA (PDB ID: 6HY7) (93). The lowest Discrete Optimized Protein Energy (DOPE) score model (94) was selected and used without any further optimization.

### 2.7 Molecular docking – docking

The nAChR ligand structures were obtained in 3D SDF format from the Protein Data Bank and were prepared for docking purposes. All ligands were imported into UCSF Chimera (95, 96), where hydrogen atoms were added, and bond orders were verified and corrected as needed. Additionally, protonation states were adjusted to reflect physiological pH (i.e, ~7.4) using Marvin (Version 17.21. from Chemaxon, https://www.chemaxon.com). Ligand preparation was completed by generating PDBQT files using AutoDockTools (ADT) in AutoDock4 (Version 4.2.6) (97). Docking was performed with the Lamarckian Genetic Algorithm (LGA) for conformational search and scoring of ligand–receptor binding poses using AutoDock4 (97). The binding site was defined by generating a grid using the AutoDock4 graphical user interface (GUI). The grid box was centered on the top of the loop C binding pocket, with box dimensions of 60 × 60 × 60 Å, and expanded to encompass adjacent regions, allowing exploration of additional potential binding pockets. All rotatable bonds of the ligands were treated as flexible while the receptor was kept rigid. Finally, conformations with the most favorable free energy of binding were selected for analyzing the interactions between the target receptor and the ligands.

The electrostatic surface potential for complementary subunits was calculated using the Adaptive Poisson–Boltzmann Solver (APBS, Version 3.4.1) (98–101). Ligand interaction diagrams were generated using the Protein–Ligand Interaction Profiler (PLIP, https://plip-tool.biotec.tu-dresden.de/) (102, 103). PyMOL was used for 3D molecular graphics, structural alignments and visualizations.

## 3 Results

### 3.1 The putative α7-selective nAChR agonists S24795 and PNU-282987 inhibit the BzATP-mediated IL-1β release

**Figure 2:**
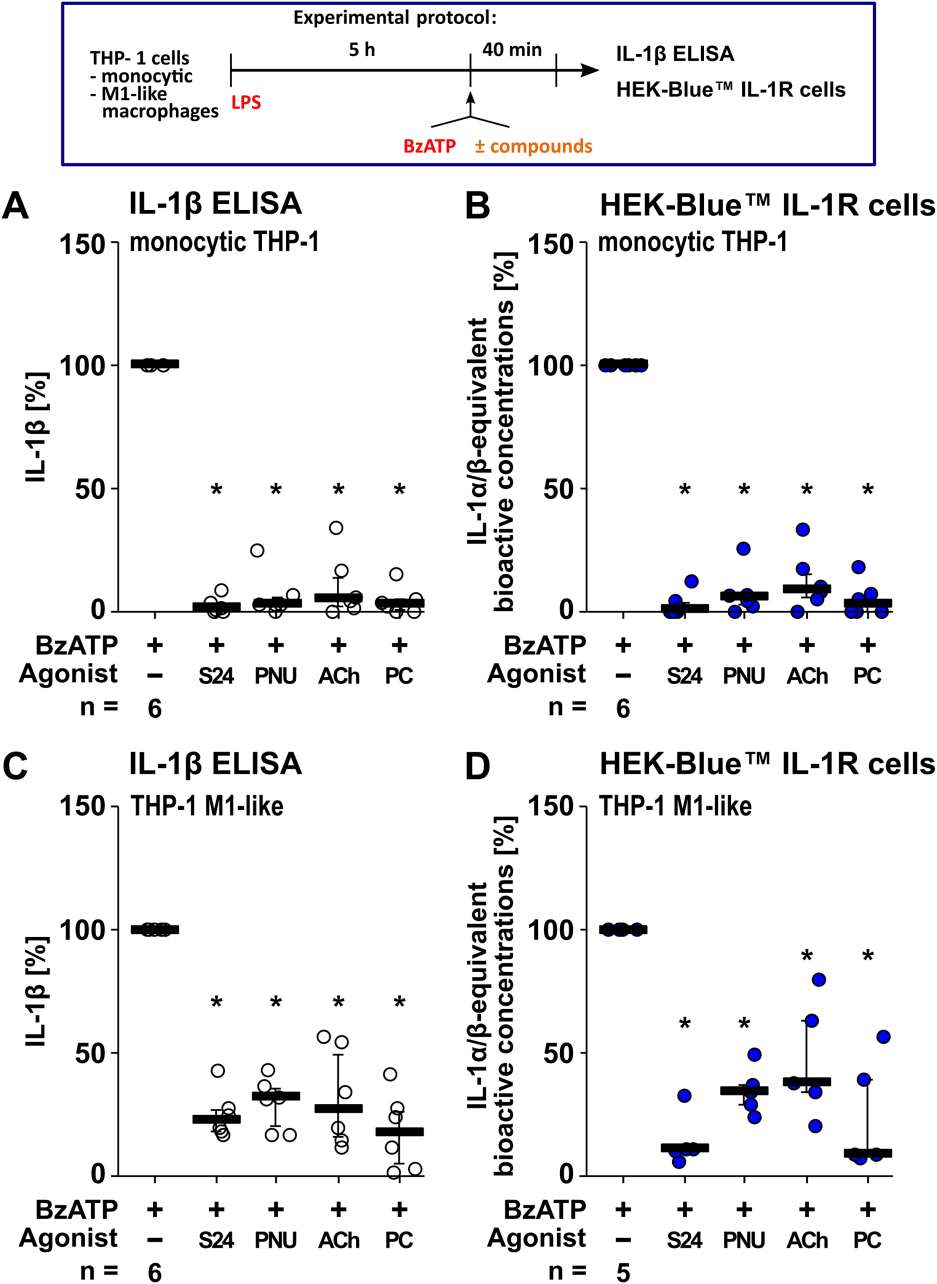
The putative α7-selective nicotinic acetylcholine receptor (nAChR) agonists S24795 (S24) and PNU-282987 (PNU) inhibit the BzATP-mediated interleukin (IL)-1β release. Monocytic THP-1 cells and THP-1 cell-derived M1-like macrophages were primed for 5 h with lipopolysaccharide (LPS; 1 µg/ml). The P2X7 receptor agonist BzATP was added for another 40 min to trigger IL-1β release, which was quantified by ELISA **(A,C)** and by measuring IL-1α/β-equivalent bioactive concentrations using HEK-Blue™ IL-1R cells in a QUANTI-Blue™ assay **(B, D)**. The BzATP- (100 µM) induced release of IL-1β was investigated in the presence and absence of the nAChR agonists S24 (50 µM) and PNU (10 µM), acetylcholine (ACh, 10 µM) or phosphocholine (PC, 200 µM). The amount of IL-1β released in response to BzATP was calculated by subtracting the IL-1β concentrations of cells treated with LPS alone. In each experiment, the IL-1β concentrations obtained after stimulation with BzATP were set to 100% and all other values were calculated accordingly. Data are presented as individual data points, bars represent median, whiskers percentiles 25 and 75. *p ≤ 0.05, different from LPS-primed cells stimulated with BzATP alone. Friedman test followed by the Wilcoxon signed-rank test.

To test if the putative α7-selective nAChR agonists S24795 and PNU-282987 inhibit the ATP-induced IL-1β release, human monocytic THP-1 cells and THP-1 cell-derived M1-like macrophages were primed for 5 h with LPS (1 µg/ml). Thereafter, the P2RX7 agonist BzATP (104) or the pore-forming toxin nigericin (84) was applied for 40 min to trigger IL-1β release. The released IL-1β was quantified in cell culture supernatants by both human IL-1β ELISA and by measuring IL-1α/β-equivalent bioactive concentrations using HEK-Blue™ IL-1R cells in a QUANTI-Blue™ assay (**Figure 2**). A comparison of the calculated IL-1β concentrations using either the IL-1β ELISA or the HEK-Blue™ IL-1R cell assay can be found in **Supplementary Figure S1**.

LPS-priming alone did not induce the release of a relevant amount of IL-1β (monocytic THP-1 cells: 1 to 10 pg/ml; THP-1 cell-derived M1-like macrophages: 4 to 27 pg/ml; each n = 6) as measured by ELISA. As expected (39, 75, 83), the BzATP-mediated release of IL-1β by monocytic THP-1 cells and THP-1 cell-derived M1-like macrophages was significantly (p ≤ 0.05; n = 6) inhibited in the presence of ACh and PC (**Figures 2A, C**), and, interestingly, by the putative α7-selective nAChR agonist S24795 and PNU-282987 (p ≤ 0.05; n = 6; **Figures 2A, C**). Cell death in monocytic THP-1 cells and THP-1 cell-derived M1-like macrophages as estimated by the LDH activity in cell culture supernatants remained low (monocytic THP-1 cells max. LDH 4.3 ± 2.4%; THP-1 cell derived M1-like macrophages max. LDH 15.2 ± 3.3%; **Supplementary Table S1**).

Moreover, S24795 and PNU-282987 had no impact (p ≥ 0.05; n = 6) on the ATP-independent release of IL-1β, as tested by using nigericin (50 µM; added together with 0.5 U/ml apyrase) instead of BzATP (**Supplementary Figure S2**). The cell death in monocytic THP-1 cells as estimated by the LDH activity in cell culture supernatants remained low (max. LDH 13.8 ± 2.5%; **Supplementary Table S2**).

### 3.2 The agonistic effects of S24795, PNU-282987, ACh and PC are sensitive to inhibition by MLA

**Figure 3:**
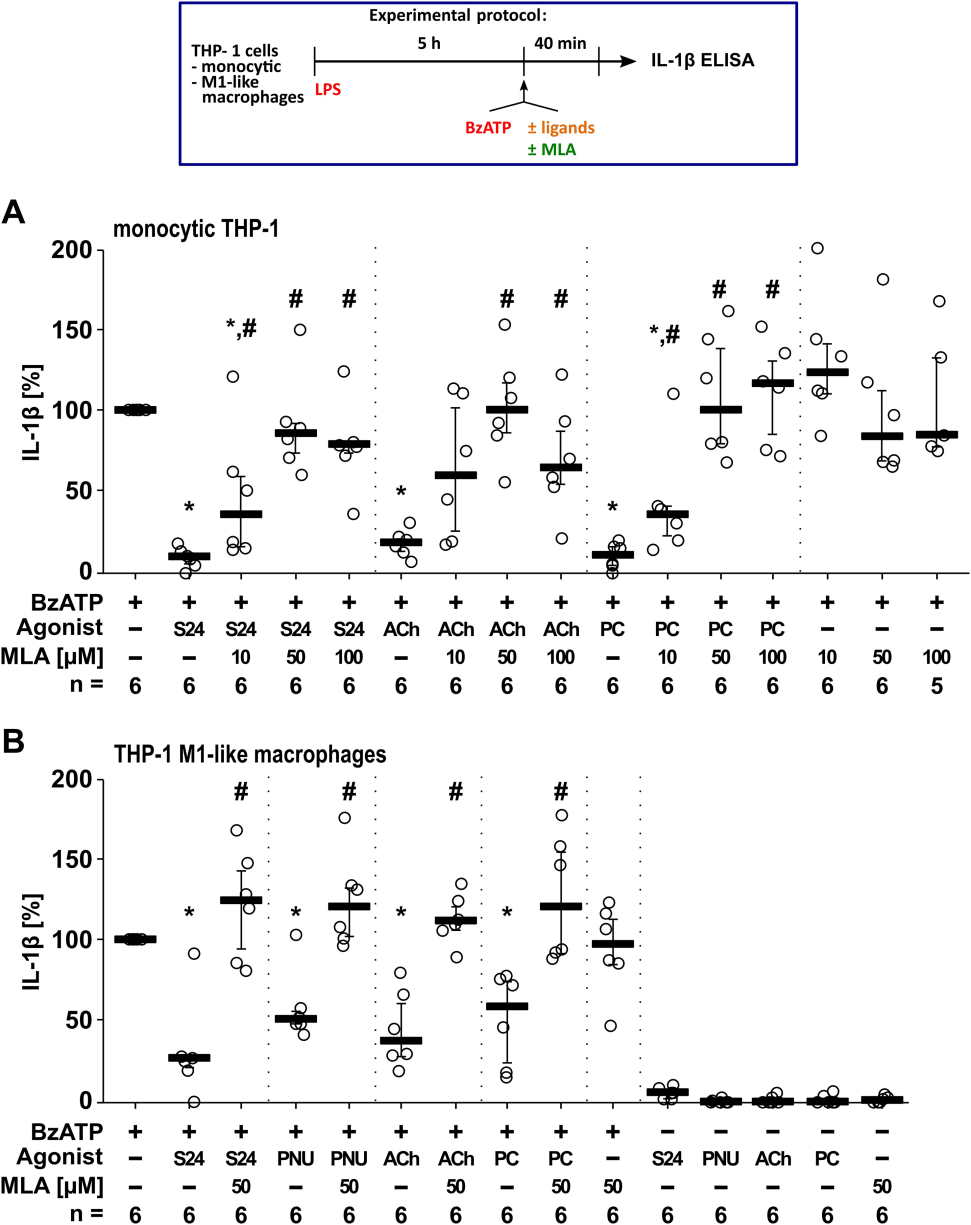
The effect of α7 nicotinic acetylcholine receptor (nAChR) agonists S24795 (S24) and PNU-282987 (PNU) on the BzATP-mediated release of interleukin (IL)-1β is sensitive to methyllycaconitine (MLA). Monocytic THP-1 cells **(A)** and THP-1 cell-derived M1-like macrophages **(B)** were primed for 5 h with LPS (LPS; 1 µg/ml). The P2X7 receptor agonist BzATP was added for another 40 min to trigger IL-1β release, which was measured by ELISA. The BzATP- (100 µM) induced release of IL-1β was investigated in the presence and absence of the nAChR agonists S24 (50 µM) and PNU (10 µM), or the ligands acetylcholine (ACh, 10 µM) and phosphocholine (PC, 200 µM). In addition, MLA was co-applied. The amount of IL-1β released in response to BzATP was calculated by subtracting the IL-1β concentrations measured in supernatants of cells treated with LPS alone. In each experiment, the IL-1β concentrations obtained after stimulation with BzATP were set to 100% and all other values were calculated accordingly. Data are presented as individual data points, bars represent median, whiskers percentiles 25 and 75. *p ≤ 0.05, different from LPS-primed cells stimulated with BzATP alone; #p ≤ 0.05, different from LPS-primed cells stimulated with BzATP plus an agonist. Friedman test followed by the Wilcoxon signed-rank test.

To test the effect of MLA on ATP-mediated release of IL-1β, human monocytic THP-1 cells were primed for 5 h with LPS (1µg/ml). Stimulation of LPS-primed cells with BzATP resulted in elevated IL-1β levels in supernatants of monocytic THP-1 cells in the range of 34 to 100 pg/ml (n = 6). As expected (39, 83), BzATP-mediated release of IL-1β was significantly (p ≤ 0.05; n = 6) inhibited in the presence of ACh (10 µM) or PC (200 µM; **Figure 3A**). Similar results were found for the putative α7-selective nAChR agonist S24795 (50 µM; p ≤ 0.05; n = 6; **Figure 3A**). Previously, we showed that the anti-inflammatory effect of ACh and PC depends on nAChR subunits α7, α9 and α10 (12, 41, 42), the inhibitory effect of all tested agonists was reversed in the presence of MLA in a concentration-dependent manner (n = 6; **Figure 3A**). MLA alone had no impact on BzATP-induced release of IL-1β (**Figure 3A**). The viability of the cells was not affected by the agonists and MLA, as measured by LDH activity (**Supplementary Table S3**).

We further tested BzATP-induced release of IL-1β by THP-1 cell-derived M1-like macrophages (**Figure 3B**). Priming of M1-like macrophages for 5 h with LPS induced IL-1β release ranging from 6 to 102 pg/ml (n = 6). In response to BzATP, M1-like macrophages released IL-1β in the range of 324 to 1265 pg/ml (n = 6). Like in monocytic cells, the BzATP-mediated release of IL-1β was significantly (p ≤ 0.05; n = 6) inhibited in the presence of ACh or PC, as well as the α7-selective agonists S24795 and PNU-282987 (**Figure 3B**). MLA (50 µM) reversed the inhibitory effect of these agonists (**Figure 3B**). The LDH was slightly increased in LPS-primed cells that were stimulated with BzATP (from 8.5 ± 2.7% to 22.9 ± 4.6%; n = 12) likely due to their metabolically active state that makes them highly susceptible to secondary stimuli (like ATP), leading to rapid, increased membrane permeability and subsequent LDH release (105). However, the viability of the cells was not affected by the agonists and MLA, as estimated by measuring LDH activity (**Supplementary Table S3**). Only samples treated with PNU-282987 showed slightly increased LDH values (**Supplementary Table S3**). However, since PNU-282987 significantly inhibited the BzATP-induced release of IL-1β (**Figure 3B**), it can be expected that PNU-282987-induced cell death is not relevant.

In summary, the putative α7-selective nAChR agonists as well as ACh and PC efficiently inhibit ATP-induced release of IL-1β and the activated nAChRs are all blocked by MLA (**Figure 3**).

### 3.3 The effect of the α7 nAChR agonists S24795 and PNU-282987 are sensitive to inhibition by α-conopeptides [V11L;V16D]ArIB and RgIA4

**Figure 4:**
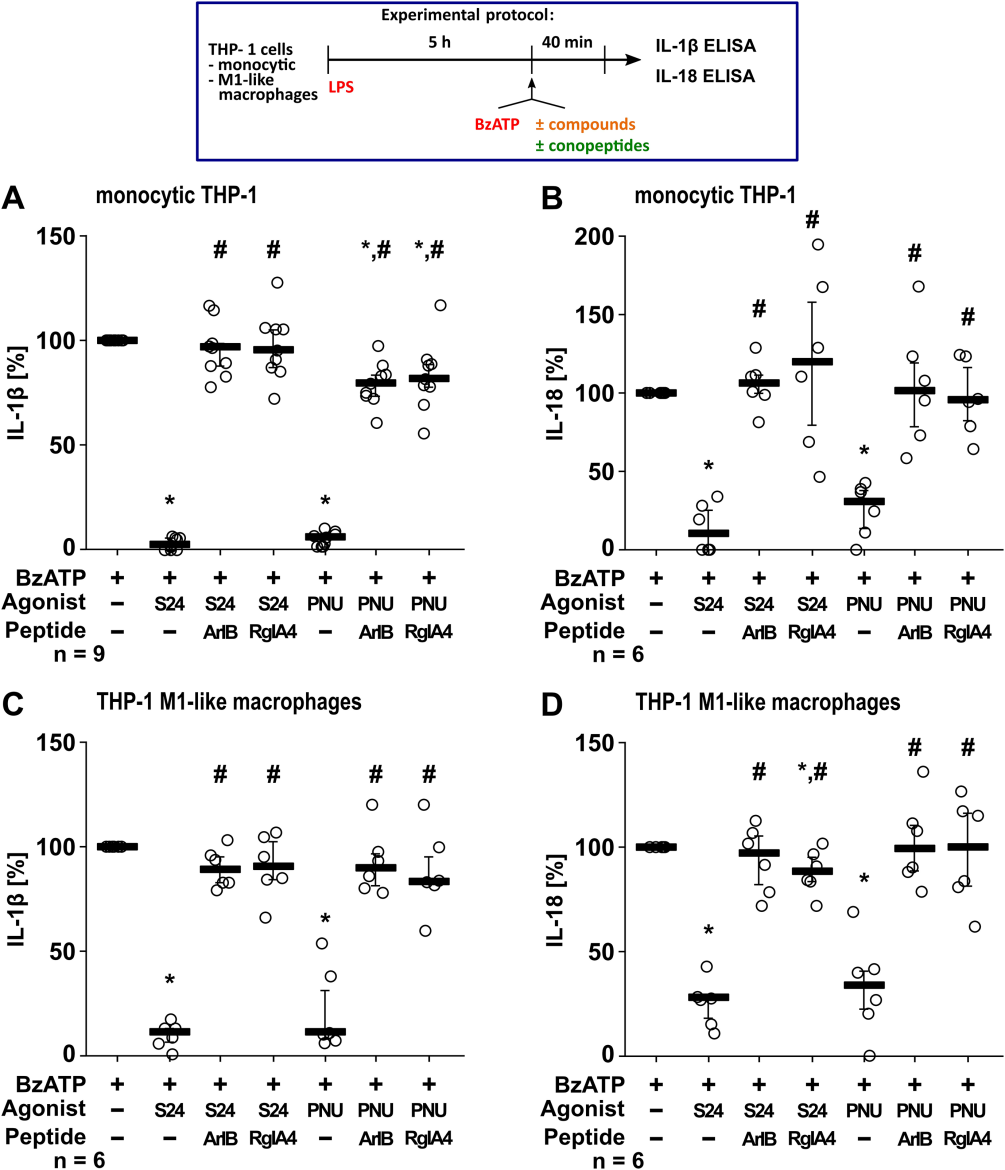
The effect of the putative α7-selective nicotinic acetylcholine receptor (nAChR) agonists S24795 (S24) and PNU-282987 (PNU) on the BzATP-mediated cytokine release is sensitive to the α-conopeptides [V11L,V16D]ArIB and RgIA4. Monocytic THP-1 cells and THP-1 cell-derived M1-like macrophages were primed for 5 h with LPS (LPS; 1 µg/ml). The P2X7 receptor agonist BzATP was added for another 40 min to trigger the release of IL-1β **(A, C)** and IL-18 **(B, D)**, which was measured by ELISA. The BzATP- (100 µM) induced release of IL-1β and IL-18 was investigated in the presence and absence of the α7 nAChR agonists S24 (50 µM) and PNU (10 µM). To test for the involvement of nAChR subunits the conopeptides Rg1A4 (RgIA; 200 nM) or [V11L,V16D]ArIB (500 nM) were co-applied. The amount of IL-1β and IL-18 released in response to BzATP was calculated by subtracting the IL-1β concentrations measured in supernatants of cells treated with LPS alone. In each experiment, the IL-1β concentrations obtained after stimulation with BzATP were set to 100% and all other values were calculated accordingly. Data are presented as individual data points, bars represent median, whiskers percentiles 25 and 75. *p ≤ 0.05, different from LPS-primed cells stimulated with BzATP alone; #p ≤ 0.05, different from LPS-primed cells stimulated with BzATP plus an agonist. Friedman test followed by the Wilcoxon signed-rank test.

To further test the involvement of nAChR subunits, the α-conopeptides [V11L;V16D]ArIB (specifically antagonizing α7 nAChR) (76–78) and RgIA4 (antagonizing nAChRs composed of subunits α9 and α9α10) (79–81) were used (**Figure 4**). Experiments were performed on monocytic THP-1 cells (**Figures 4A, B**) and THP-1 cell-derived M1-like macrophages (**Figures 4C, D**). We have previously shown that transcripts of the nAChR subunits α7, α9, and α10 are expressed by human monocytic THP-1 cells and THP-1 cell-derived macrophages (75). BzATP-mediated release of IL-1β (**Figures 4A, C**) and IL-18 (**Figures 4B, D**) by LPS-primed cells was significantly inhibited in the presence of S24795 and PNU-282987 (p ≤ 0.05; n = 6). Interestingly, S24795 and PNU-282987 were sensitive to both conopeptides, indicating that α7, α9 and/or α10 subunits were required to mediate the inhibitory effect on the BzATP-induced release of IL-1β (**Figure 4**). The viability of the cells was not affected by the agonists and conopeptides, as measured by LDH activity (**Supplementary Table S4**). These data indicate that the putative α7 nAChR agonists S24795 and PNU-282987 are sensitive to the α-conopeptides [V11L;V16D]ArIB and RgIA4.

To verify the specificity of RgIA4, *Xenopus laevis* oocytes heterologously expressing human homomeric α7 or homomeric α9 nAChRs were subjected to TEVC measurements. While RgIA4 (200 nM) inhibited the ACh-induced current responses of α9 nAChRs (p ≤ 0.05; n = 7), no impact was seen in α7 nAChR-expressing oocytes (**Supplementary Figure S3**).

### 3.4 S24795 and PNU-282987 interact with the α9* nAChR subunits

**Figure 5:**
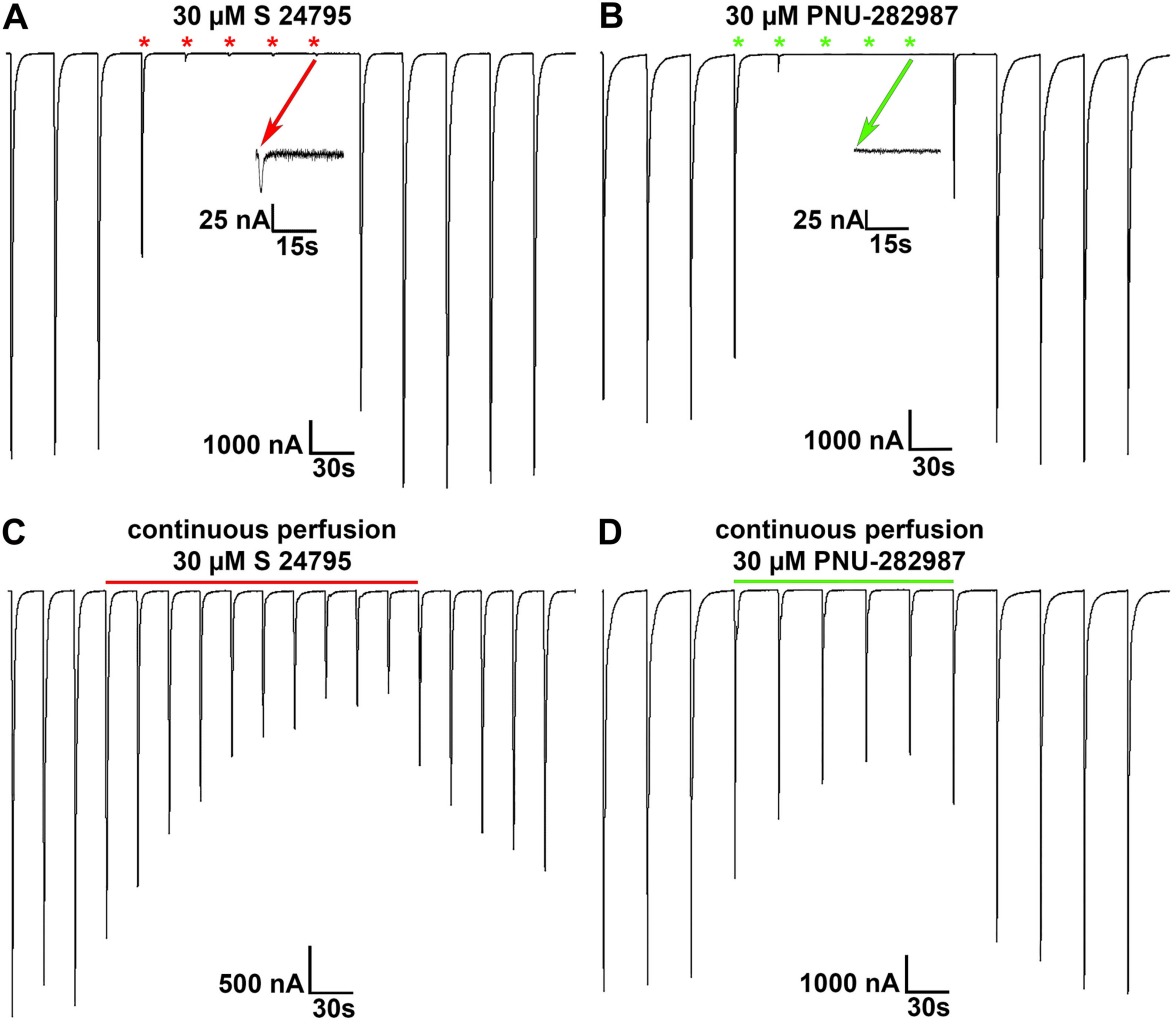
The putative α7-selective agonists S24795 and PNU-282987 interact with α9α10 nAChRs. *Xenopus laevis* oocytes heterologously expressing human α9α10 nicotinic acetylcholine receptor (nAChR) were subjected to two-electrode voltage-clamp experiments and exposed to the α7-selective agonists S24795 or PNU-282987. **(A, B)** Oocytes expressing α9α10 nAChRs were stimulated with 1 s pulses of acetylcholine (ACh; 60 µM) until steady baseline responses were observed, then stimulated with S24795 **(A)** or PNU-282987 **(B)** and the responses compared to those evoked by ACh. The asterisks indicate 1 s pulses of S24795 (red) or PNU-282987 (green). **(C, D)** S24795 and PNU-282987 were also tested for antagonist activity by perfusion of the compounds during stimulation with ACh. Oocytes were stimulated with 1 s pulses of ACh until steady baseline responses were observed, then the control solution was switched to one containing S24795 **(C)**; 10 min perfusion indicated by the horizontal red bar above the current traces) or PNU-282987 **(D)**; 5 min perfusion, horizontal green bar). Representative current traces are shown **(A-D)** from 5–8 oocytes each.

To further investigate if S24795 and PNU-282987 can directly interact with α9* nAChRs, we next performed electrophysiological TEVC measurements on *Xenopus laevis* oocytes heterologously expressing human α9α10 nAChRs. Oocytes expressing α9α10 nAChRs were stimulated with 1 s pulses of ACh (60 µM) until steady baseline current-responses were observed (**Figure 5**). To test for an agonist-like function of S24795 and PNU-282987, oocytes were then stimulated with 1 s pulses of the ligands. S24795 (30 µM) evoked small responses (see inset for an expanded current trace) that were 0.5 ± 0.2% (mean ± SD; n = 5) of the responses evoked by ACh (**Figure 5A**). PNU-282987 (30 µM) failed to evoke detectable currents (**Figure 5B**). The responses from stimulation with PNU-282987 were 0.3 ± 0.3% (n = 5) of the responses evoked by ACh. Note that upon switching from ACh pulses to pulses of S24795 (**Figure 5A**) or PNU-282987 (**Figure 5B**), approximately three 1 s pulses were required to eliminate residual ACh from the perfusion lines.

Next, S24795 and PNU-282987 were tested for antagonist activity by constant perfusion of the compounds during stimulation with ACh. For this purpose, oocytes expressing α9α10 nAChRs were stimulated with 1 s pulses of ACh until steady baseline responses were observed, then the control solution was switched to one containing S24795 or PNU-282987 (30 µM each). In the presence of S24795 the ACh-evoked current amplitudes were reduced to 22 ± 9% (n = 5) of control values (ACh alone; **Figure 5C**). PNU-282987 reduced the ACh-evoked current amplitudes to 40 ± 21% (n = 8; **Figure 5D**).

### 3.5 Molecular docking analysis reveal an interaction of S24795 and PNU-282987 with α9 and α10 nAChR subunits

**Figure 6:**
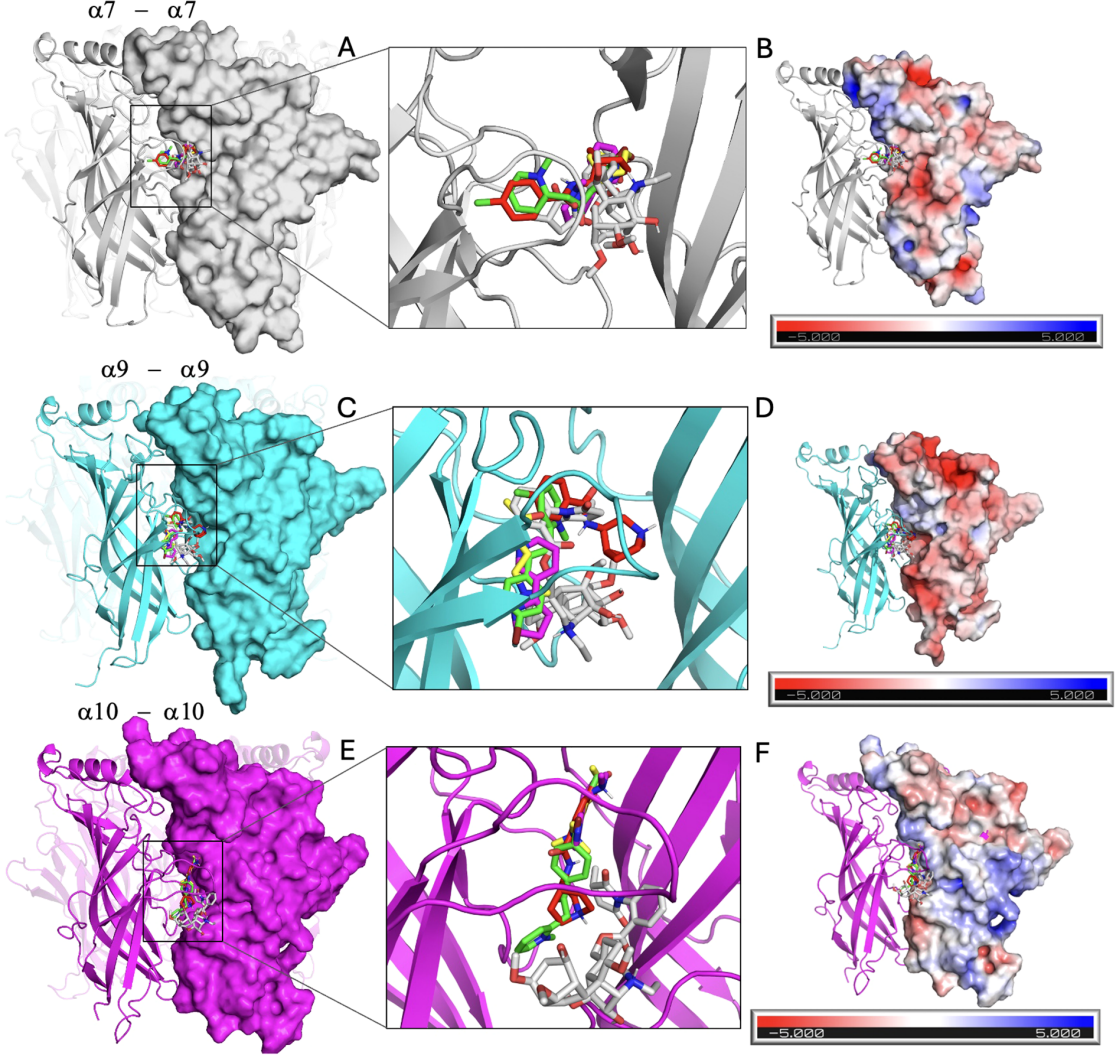
Cartoon highlighting the extracellular domain with two chains of α7, α9, and α10 of homomeric nicotinic acetylcholine receptor (nAChR). Docked ligands in the α7 [shown in grey; **(A, B)**], α9 [shown in cyan; **(C, D)**], and α10 [shown in pink; **(E, F)**] nAChR are shown. Docked poses of representative compounds S24795 (green), PNU-282987 (red), methyllycaconitine (MLA) (grey), acetylcholine (yellow), and nicotine (Pink), demonstrate binding within the C-loop region and its vicinity. Electrostatic surface potentials for complementary chain of a7, a9 and a10 are displayed in **(B, D, F)** respectively.

Crystal and cryogenic electron microscopy (cryo-EM) structures revealed that nAChRs are organized as pentameric assemblies of homologous subunits arranged around a central ion-conducting pore (5, 106). The ligand-binding site is located at the interface of two adjacent subunits within the ECD, primarily formed by six conserved loops (A–F) (**Supplementary Figure S4**). To gain an insight about the binding modes of the putative α7-selective agonists S24795 and PNU-282987, MLA, and the classical nAChR ligands ACh and nicotine on an atomistic perspective of ligand-protein interactions, molecular docking was performed. The crystal structure of the homomeric human α7 (PDB ID: 7EKI) and a modeled pentameric assembly of the homomeric α9 ECD (PDB ID: 6HY7) were used. The homomeric α10 nAChR ECD was generated by homology modeling using the PDB ID 6HY7 as the template. All compounds were docked into the binding sites, and the grid box was expanded to encompass adjacent regions to explore additional potential binding pockets. The resulting ligand–receptor complexes are shown in **Figure 6**.

Docking analysis revealed that all compounds consistently bound within the C-loop region of the α7 nAChR, corresponding to the orthosteric binding pocket. The docking results reveal distinct binding preferences of the tested ligands for the homomeric α7 and, interestingly, α9 and α10 nAChRs ECD (**Table 2**). Among all the compounds, the putative α7-selective agonists S24795 and PNU-282987 exhibited the most favorable binding energies, with S24795 showing the strongest affinity for both α7 (–5.9 kcal/mol), α9 (–6.1 kcal/mol) and α10 (–5.2 kcal/mol; **Table 2**). These observations suggest that S24795 could be well-suited to interact with conserved structural features of the orthosteric site in all receptor subunits. PNU-282987 also demonstrated consistently high docking scores (–5.1 and –5.4 kcal/mol; **Table 2**). MLA had the highest docking score at homomeric α9 nAChR (–6.2 kcal/mol; **Table 2**).

**Table 2:** Binding affinities (kcal/mol) for selected ligands on human homomeric α7, α9 and α10 nicotinic acetylcholine receptors (nAChRs) obtained using Autodock 4.

Ligand	α7	α9	α10
Acetylcholine	-2.9	-2.4	-2.2
Methyllycaconitine	-5.5	-6.2	-4.8
Nicotine	-3.4	-3.9	-3.8
PNU-282987	-5.1	-5.4	-5.4
S24795	-5.9	-6.1	-5.2

**Figure 7:**
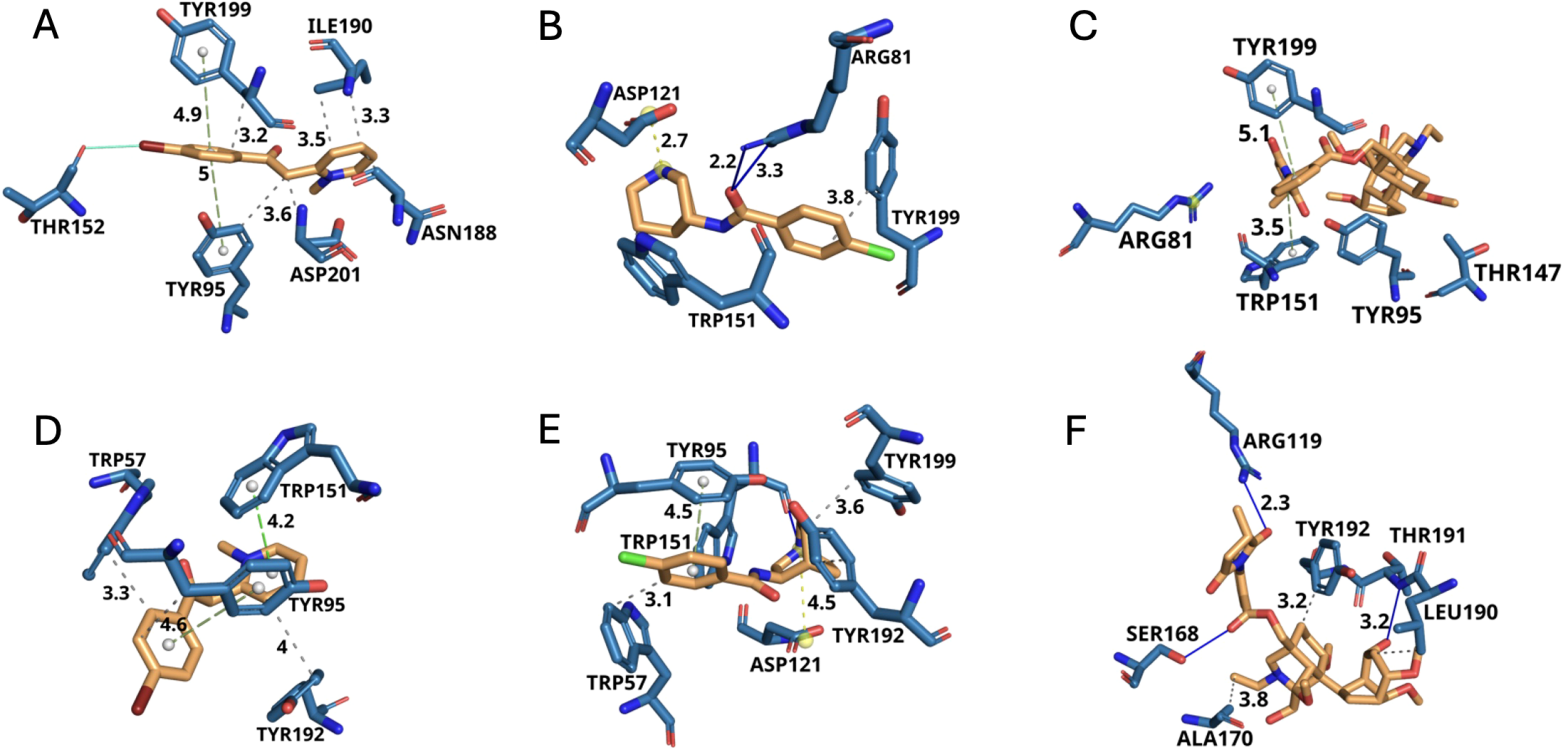
Binding mode and ligand interaction diagram for putative α7-selective nicotinic acetylcholine receptor (nAChR) agonists in the extracellular domain of homomeric α9 and α10 nAChRs. Ligand interaction diagrams for S24795 **(A, D)**, PNU-282987 **(B, E)**, and methyllycaconitine (MLA; **(C, F)** in the α9 **(A–C)** and the α10 **(D-F)** nAChR subunit. Ligand and residues are shown in sticks and distances and interactions are shown in lines: blue lines indicate hydrogen bonds, gray dotted lines indicate hydrophobic interactions, yellow dotted lines indicate salt-bridges interactions, green dotted lines indicate (π-stacking or π-cation), and green lines represent halogen-bonds interaction.

Similar to ACh and nicotine (**Supplementary Figure S5**), the putative binding modes and interaction analyses of S24795, PNU-282987, and MLA with the homomeric α9 nAChR reveal well-stabilized complexes within the orthosteric site. S24795 forms multiple hydrophobic interactions with Tyr95 (Loop A), Asn188, Ile190, Tyr199 (TryC2), and Asp201, complemented by π-stacking with Tyr95 (Loop A), and Tyr199 (TryC2), as well as a directional halogen interaction with Thr152, which together reinforce the ligand’s orientation and aromatic complementarity within the binding pocket (**Figure 7A**). PNU-282987 engages the receptor through a hydrogen bond with Arg81, along with hydrophobic contacts involving Trp151 (Loop B) and Tyr199, while a salt bridge with Asp121 further stabilizes the ligand via electrostatic interactions, collectively supporting efficient binding (**Figure 7B**). Similarly, MLA is accommodated through a combination of π-stacking, π-cation, and hydrophobic interactions, ensuring stable anchoring across the binding site. Overall, all three ligands exploit a consistent network of hydrophobic, aromatic, and polar interactions, highlighting the orthosteric site’s capacity to support diverse stabilizing forces and well-oriented ligand binding in α9 (**Figure 7C**).

The α10 nAChR engages the ligands through a similar and consistent network of hydrophobic, aromatic, and polar interactions that define a well-structured binding environment. Comprehensive illustrations of the binding modes for ACh and nicotine are provided in **Supplementary Figure S5**. Hydrophobic residues such as Trp57 (Loop D), Tyr95, Tyr192 (TryC1), Leu190, and Ala170 create a nonpolar pocket accommodating the aromatic and aliphatic features of the ligands, while aromatic residues, particularly Tyr95 (Loop A) and Trp151 (Loop B), contribute through π-stacking interactions that stabilize S24795 and PNU-282987 (**Figures 7D, E**). MLA, by contrast, relies more heavily on a complementary network of hydrogen bonds involving Arg119, Ser168, and Thr191, which anchor the ligand across both principal and complementary subunits (**Figure 7F**). Although each ligand interacts with the pocket in distinct ways, all three ligands in α10 depend on a shared combination of hydrophobic packing, aromatic stacking, and strategic hydrogen bonding, collectively defining the characteristic interaction landscape of this receptor and highlighting parallels in ligand recognition between α9 and α10.

## 4 Discussion

This study advances the current understanding of the CAS by demonstrating that ligands historically categorized as α7-selective nAChR modulators functionally engage α9* nAChRs in human mononuclear phagocytes. Our results show that putative α7-specific agonists (S24795, PNU-282987) interact with heterologously expressed human α9α10 nAChRs and efficiently inhibit the ATP-mediated release of the pro-inflammatory cytokines IL-1β/IL-1α and IL-18 by mononuclear phagocytes via unconventional α9* nAChRs (**Table 3**). The observed effects were sensitive to antagonists directed against both α7 and α9/α9α10 subunits. Our findings build upon earlier studies that have linked signaling of nAChRs containing subunits α7, α9 and α10 to the regulation of the NLRP3 inflammasome, a critical component in the control of pro-inflammatory cytokine release (12, 41, 42). By demonstrating that both α7 and α9* subunits contribute to these regulatory mechanisms, the present results support earlier studies (12, 41, 42) and refine our understanding of how nAChRs with overlapping subunit compositions function within immune cells. This provides a more nuanced framework for interpreting the roles of nAChR subtypes in immune modulation, highlighting the complexity and potential importance of α9* nAChRs in the context of inflammation and their potential as therapeutic targets for inflammatory diseases and pain.

**Table 3:** Summary. Putative α7-selective ligands (S24795, PNU-282987, MLA) interact with α9-containing nAChRs and modulate immune functions of human mononuclear phagocytes.

Ligand	Effect on ATP-induced IL-1α/IL-1β/IL-18 release	Effect on heterologously expressed nAChRs	Effect on ACh-induced ion currents	Docking analysis on homomeric α7, α9 and α10 nAChRs
S24795	Inhibition via subunits α7 and/or α9*.	Small responses that were 0.5 ± 0.2% of the responses evoked by ACh.	Reversibly inhibits ACh-induced ion currents of α9α10 nAChRs.	Strong binding affinity: α9 > α7 > α10.
PNU-282987	Inhibition via subunits α7 and/or α9*.	No ionotropic effects on α9α10 nAChRs.	Reversibly inhibits ACh-induced ion currents of α9α10 nAChRs.	Strong binding affinity: α9 ≥ α10 > α7.
MLA	Antagonizing the effect of S24795, PNU-282987, ACh and PC.	Antagonist of α7, α9 and α9α10 nAChRs (**Table 1**) (40).	Reversibly inhibits ACh-induced ion currents of α7, α9 and α9α10 nAChRs (**Table 1**) (40).	Strong binding affinity: α9 > α7 > α10.

α9*, denotes the potential presence of other nAChR subunits; ACh, acetylcholine; IL, interleukin; MLA, methyllycaconitine; nAChRs, nicotinic ACh receptors; PC, phosphocholine.

The canonical concept of the CAS was originally shaped by studies demonstrating that vagal nerve stimulation or α7 nAChR activation suppresses macrophage TNF-α production through a JAK2–STAT3-dependent mechanism (15, 16, 22, 107, 108). Subsequent studies expanded this model by showing that α7 activation interferes with NF-κB nuclear translocation and MAPK signaling in monocytes and macrophages (16, 109, 110). In previous studies we demonstrated that activation of nAChRs containing subunits α7, α9 and/or α10 in monocytic cells, macrophages, and epithelial cells down-regulate the response of the ATP-sensitive P2RX7 thereby inhibiting NLRP3-inflammasome assembly and, consequently, the release of IL-1β (12, 39, 41, 42, 75, 111, 112). Interestingly, this cholinergic control mechanism seems to be mediated by a metabotropic signaling of these unconventional nAChRs to inhibit the ionotropic function of the P2RX7 and, thus, the ATP-mediated release of IL-1β (12, 39, 41, 43, 83, 85, 113). We provided first evidence that this metabotropic signaling pathway is mediated by the activation of endothelial nitric oxide synthase (eNOS) (83). Upon activation, eNOS induces a modification of the P2RX7 within its C-terminal cytoplasmic domain which inactivates its ionotropic function. This suppresses ATP signaling and the subsequent release of IL-1β (83). This is in line with other reports that non-neuronal nAChRs can modulate innate immune signaling independently of classical ionotropic receptor functions [as summarized in (24)]. By using mononuclear phagocytes obtained from nAChR gene-deficient mice, as well as by doing siRNA experiments on human monocytes to silence the expression of nAChR subunits α7, α9 or α10 we found that the inhibition of ATP-dependent release of IL-1β by ACh, PC, choline and nicotine depends on the presence of all three subunits α7, α9 and α10 (**Table 1**). The inhibitory effect on the ATP-mediated IL-1β release by the unconventional PC-bearing agonists glycerophosphocholine (G-PC) and lysophosphatidylcholine (LPC) was shown to be dependent on nAChRs containing subunits α9 and α10, without an essential contribution of the α7 subunit (42).

Several independent studies have implicated a role of α9- and α10-containing nAChRs in inflammation and nociception. Genetic deletion or pharmacological blockade of α9* nAChRs has been shown to alter pain behaviours in models of neuropathic and inflammatory pain (40, 79, 80, 114, 115). In mononuclear phagocytes, α9* nAChRs have been linked to regulation of cytokine release (12, 35), cell migration (116), and oxidative stress (117). Moreover, the α9α10-selective conopeptide RgIA and its analogues have been shown to produce long-lasting analgesia in animal models while suppressing inflammatory mediators, suggesting that these receptors couple to long-term immunomodulatory pathways (79, 80, 115). Our findings that RgIA4-sensitive receptors mediate suppression of IL-1β and IL-18 are therefore highly consistent with these published observations and provide a cellular mechanism linking α9* receptor engagement to inflammasome control.

Previous studies have characterized PNU-282987 as a α7-selective agonist with anti-inflammatory and neuroprotective properties in models of sepsis, inflammation, and ischemic injury (118–121). Similarly, S24795 and quaternary ammonium compounds structurally related to this compound have been reported to modulate α7 nAChRs with limited activity at heteromeric neuronal receptors (52, 122). However, several studies have noted that putative α7-selective ligands can interact with α9 and α9α10 nAChRs (39, 40, 53, 123). In a previous study MLA was shown to inhibit presynaptic nAChRs of subunit composition α3/α6β2β3 on dopamine neurons highlighting already that the use of MLA as an α7-selective antagonist should be exercised with caution (124). Verbitsky et al. (69) and we provided further evidence that MLA is not exclusively selective for α7 nAChRs but also exhibits sensitivity towards α9* nAChRs (40) (**Table 1**). In addition, Zouridakis et al. provided evidence through *in silico* experiments on X-ray crystal structures that the α9 ECD can bind MLA (125). By combining cytokine-release experiments, electrophysiological studies and molecular docking, we provide evidence that the putative α7-selective agonists S24795 and PNU-282987 interact with α9* nAChRs. We show here that S24795 only evoked small current responses on heterologously expressed human α9α10 nAChRs, and PNU-282987 did not evoke detectable currents. At the same time, ACh-evoked current amplitudes were reduced in the presence of S24795 and PNU-282987 indicating functions as weak to moderate silent agonists or partial antagonists on human α9α10 nAChRs. Similar results were found before for PC (39, 41) and the PC-bearing molecules G-PC and LPC (42) on heterologously expressed human nAChRs with different combinations of the subunits α7, α9 and α10. Of note, the inhibitory effect of G-PC and LPC on the ATP-mediated release of IL-1β by mononuclear phagocytes is sensitive to RgIA4, but at best slightly attenuated by the α7-specific antagonist [V11L;V16D]ArIB (42). In summary, our results raise doubt concerning earlier publications that characterized the role of the α7 nAChR in the CAS purely by pharmacologically approaches. The role of α9* nAChRs in inflammation and nociception and the CAS could therefore have been overlooked for a long time.

Recent high-resolution cryo-EM and X-ray structures of nAChRs have revealed the conformational plasticity of the C-loop and its critical role in distinguishing agonism, partial agonism, and desensitization (106, 126, 127). Published molecular dynamics studies have shown that ligand-dependent stabilization of distinct C-loop conformations can bias receptors toward conducting or non-conducting signaling states (106, 126). Our molecular docking results, which place S24795, PNU-282987 and MLA in energetically favourable orientations within the orthosteric binding pockets of homomeric α7, α9, and α10 are consistent with these structural observations and support the idea that shared structural determinants underlie cross-subtype pharmacology. Our docking studies are, however, limited to static receptor conformations and homomeric ECD. Prior work has shown that inclusion of lipid membranes, intracellular loops, and heteromeric receptor assemblies can substantially alter predicted binding modes (126). Future studies should therefore combine long-timescale molecular dynamics simulations with cryo-EM–based heteromeric models to better capture the conformational landscape of these receptors. The ligand interaction diagrams provided here can, however, serve as a basis for future gene-modulation experiments. Site-directed mutagenesis studies of the amino acids interacting with the ligands can help to further characterize the interactions of the ligands with the receptors.

Recent reviews have highlighted the limitations of using transformed cell lines and pharmacological tools alone to define nAChR subtype function in immune cells. For example, lack of subtype-specific antibodies and overlapping ligand-binding profiles have been recognized as major challenges in the field (24, 52, 81, 128). Our study addresses these issues by combining pharmacological inhibition with subtype-selective α-conopeptides and heterologous expression systems. Nevertheless, in line with published recommendations, future work should incorporate genetic loss-of-function models (e.g., CRISPR/Cas9-mediated knockout of *CHRNA7*, *CHRNA9*, and *CHRNA10*) and studies in primary human immune cells. We still do not know the exact subunit composition [homomers, heteromers, classical pentamers at all (24, 81)] of the unconventional nAChRs containing subunits α7, α9, and/or α10 in mononuclear phagocytes. Although we have shown that putative α7-specific agonists can interact with α9* nAChRs to inhibit the ATP-mediated cytokine-release, the precise molecular mechanisms underlying this interaction remain to be elucidated. It could be that S24795 and PNU-282987 bind to both, the α7 and α9 subunits. A heteromeric nAChR α7-selective antagonists could in principle inhibit the effect of α9-selective agonists and vice versa. Furthermore, our findings are based on *in vitro* experiments using human cell lines and *Xenopus laevis* oocytes. Future studies using primary human cells and *in vivo* models will be necessary to validate and extend our results. Finally, it is important to note that the development of selective ligands for α9* nAChRs may be challenging, given the structural similarities between different nAChR subtypes. The results provided here allow the hypothesis that the cholinergic control of ATP-mediated IL-1β release by mononuclear phagocytes can only be triggered by ligand binding to the α9 nAChR subunit. This should be clarified in the future and would also have therapeutic implications.

In conclusion, when integrated with the published literature, our data support a revised model of the CAS in which α9* nAChRs play a central and previously underappreciated role alongside α7 nAChRs. Our results align with and extend earlier work on α7-dependent immunosuppression, α9*-mediated pain and inflammation, and structural determinants of nAChR ligand recognition. This work highlights the need for more refined pharmacological and genetic tools and provides a strong rationale for targeting both α7 nAChRs and α9* nAChRs in the treatment of inflammatory diseases and pain. By understanding the complex interplay between different nAChR subtypes and their role in regulating the CAS, we can pave the way for the development of novel therapeutic strategies for inflammatory diseases and pain.

## Data Availability

The original contributions presented in the study are included in the article/**Supplementary Material**. Further inquiries can be directed to the corresponding authors.
