# Supplementary material for: Putative α7-selective ligands interact with α9-containing nicotinic acetylcholine receptors and modulate immune functions of human mononuclear phagocytes
Source: Front Immunol. 2026 Mar 11;17:1773637. doi: 10.3389/fimmu.2026.1773637 (PMC13016199; doi:10.3389/fimmu.2026.1773637)
Supplement: Supplementary file 1 [file DataSheet1.pdf]

Supplementary Material

1 Supplementary Figures and Tables

1.1 Supplementary Figures

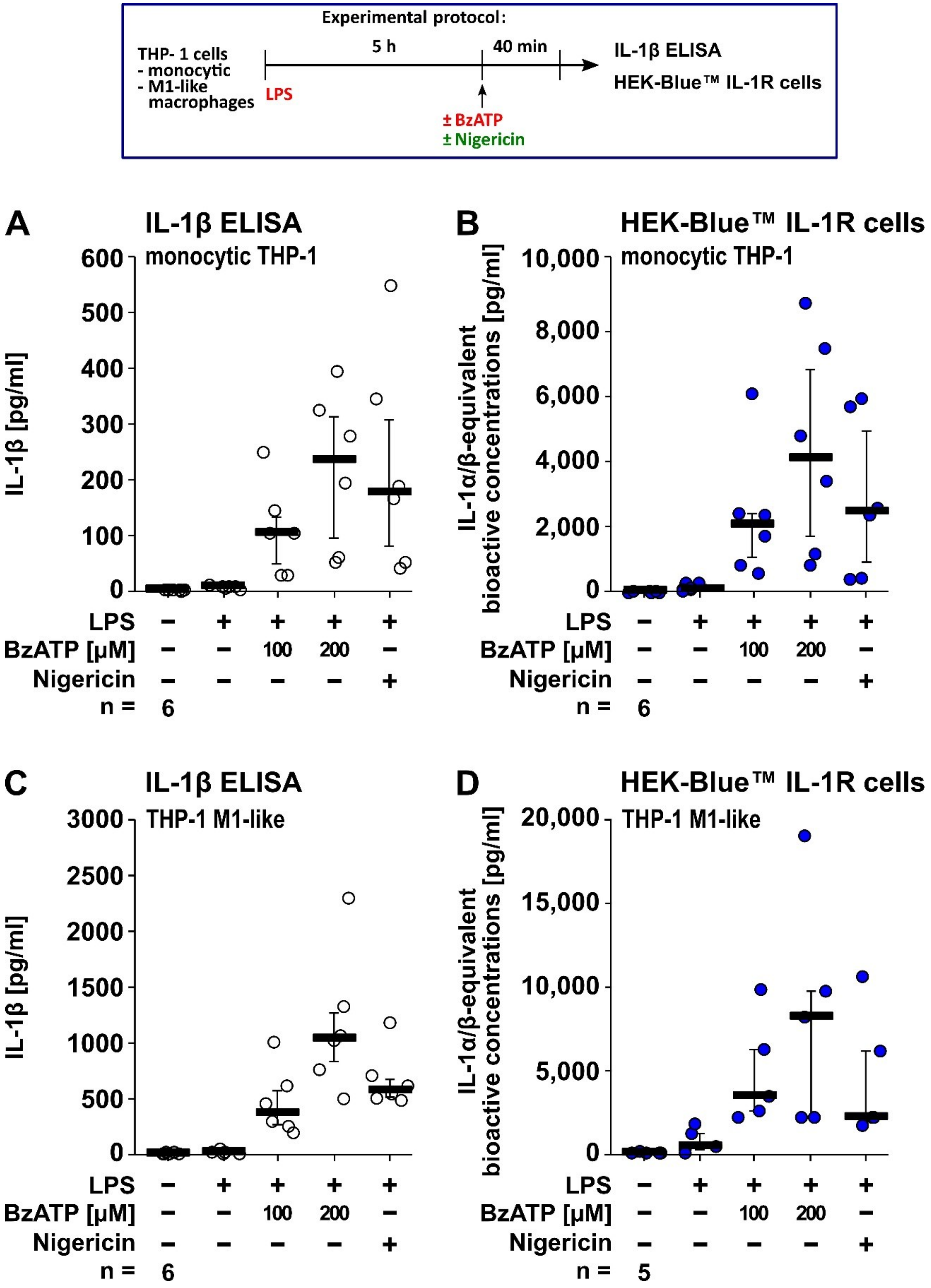

Supplementary Figure S1: Quantification of interleukin (IL)-1β in cell culture supernatants by IL-1β ELISA and IL-1α/β responsive NF-kB/AP1-SEAP reporter assay. Monocytic THP-1 cells and THP-1 cell-derived M1-like macrophages were left untreated or primed for 5 h with

lipopolysaccharide (LPS; 1 µg/ml). Thereafter, the P2X7 receptor agonist BzATP (100 µM, 200 µM) or the pore-forming toxin nigericin (added together with 0.5 U/ml apyrase) was added for another 40 min to trigger IL-1β release, which was quantified by ELISA (**A,C**) and by measuring IL-1α/β-equivalent bioactive concentrations using HEK-Blue™ IL-1R cells in a QUANTI-Blue™ assay (**B,D**).

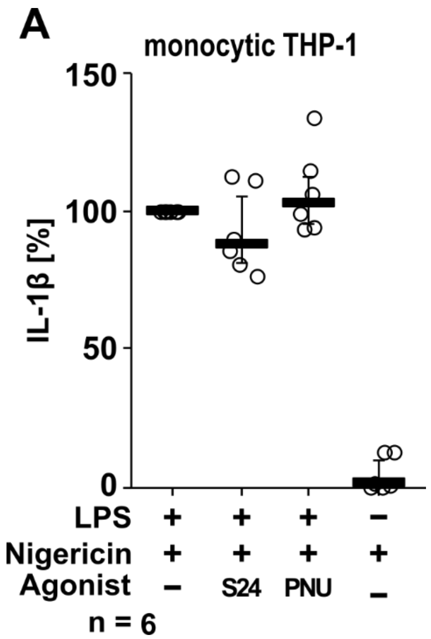

Supplementary Figure S2. The purported α7-selective nicotinic acetylcholine receptor (nAChR) agonists S24795 (S24) and PNU-282987 (PNU) have no impact on the nigericin-induced release of IL-1β by human monocytic THP-1 cells. Monocytic THP-1 cells were primed with LPS (1 µg/ml) for 5 h, and nigericin (50 µM) was added for another 40 min to trigger IL-1β release, which was measured by ELISA. S24 (50 µM) and PNU (10 µM) had no impact on the nigericin-induced release of IL-1β. Data are presented as individual data points, bars represent median, whiskers percentiles 25 and 75. Friedman test followed by the Wilcoxon signed-rank test.

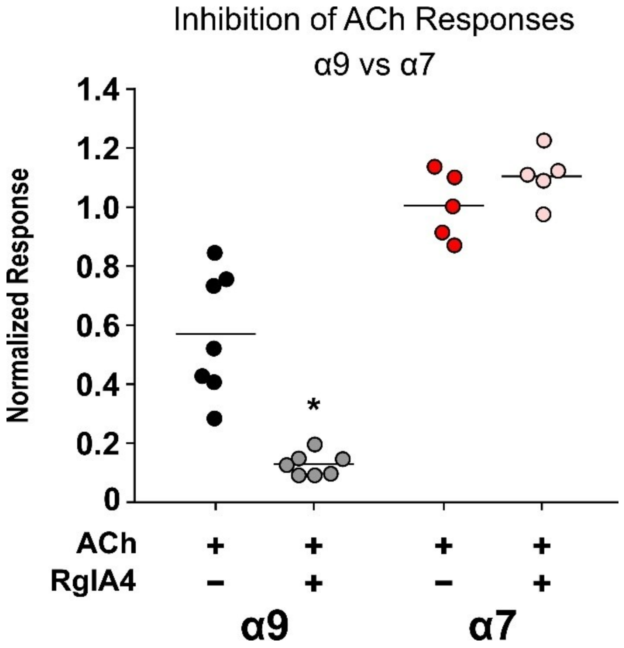

Supplementary Figure S3: The α-conopeptide RgIA4 does not inhibit acetylcholine- (ACh) induced currents on human α7 nicotinic ACh receptors (nAChRs). *Xenopus laevis* oocytes heterologously expressing human α9 or α7 nAChRs were subjected to two-electrode voltage-clamp experiments and exposed to ACh (60 µM) in the absence and presence of RgIA4 (200 nM). Data on individual responses (two responses from each of 5 – 7 cells), black bars are the averages of the replicates. *$p \leq 0.05$, significantly different from corresponding experiments with ACh alone; Kruskal–Wallis test followed by the Mann–Whitney U test.

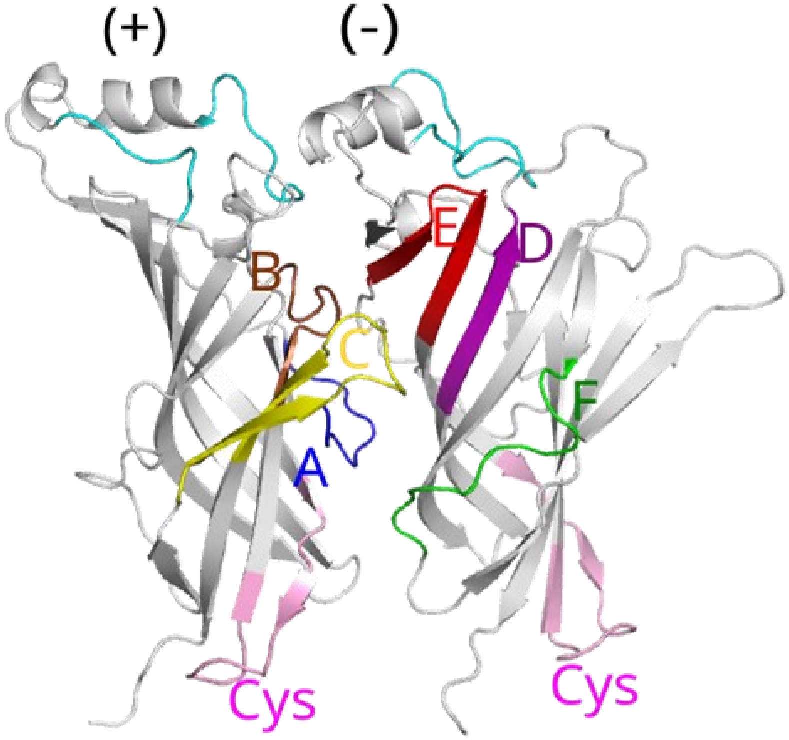

Supplementary Figure S4: Cartoon of the structural annotated extracellular domain of nicotinic acetylcholine receptor (nAChR) subunits showing the principal (+) and compensatory (-) subunit. The ligand-binding pocket is shown with the principal binding loops (A–F) and the cys-loop (Cys) indicated.

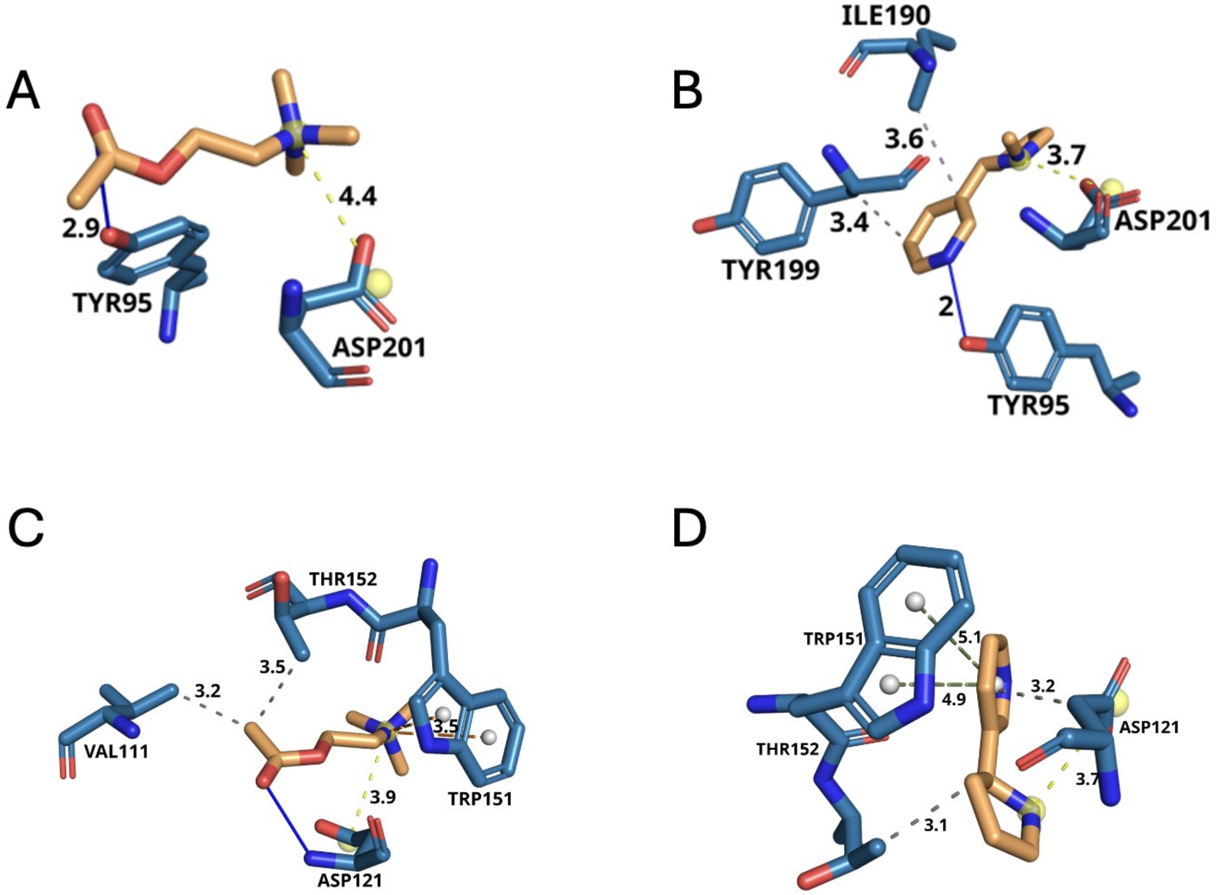

Supplementary Figure S5: Binding mode and ligand interaction diagram for acetylcholine (ACh) and nicotine in the extracellular domain of homomeric α9 and α10 nicotinic acetylcholine receptors (nAChRs). Ligand interaction diagrams for ACh (**A, C**) and nicotine (**B, D**) in the α9 (**A-B**) and the α10 (**C-D**) nAChR subunit. Ligand and residues are shown in sticks and distances and interactions are shown in lines: blue lines indicate hydrogen bonds, gray dotted lines indicate hydrophobic interactions, yellow dotted lines indicate salt-bridges interactions, green dotted lines indicate (π-stacking or π-cation), and green lines represent halogen-bonds interaction.

1.2 Supplementary Tables

Supplementary Table S1: Cell death in monocytic THP-1 cells and THP-1 cell-derived M1-like macrophages as estimated by the lactate dehydrogenase (LDH) activity in cell culture supernatants.

THP-1 cells	Treatment	Cell death [%] mean ± SD	n
**monocytic**	-	3.3 ± 2.3	6
	LPS	3.3 ± 2.3	6
	LPS, BzATP ± DMSO	3.0 ± 2.4	12
	LPS, BzATP, S24795 50 µM	2.7 ± 1.2	6
	LPS, BzATP, PNU-282987 10 µM	4.3 ± 2.4	6
	LPS, BzATP, ACh 10 µM	3.7 ± 2.0	6
	LPS, BzATP, PC 200 µM	3.8 ± 2.1	6
**M1-like**	-	8.7 ± 3.3	6
	LPS	5.3 ± 1.0	6
	LPS, BzATP ± DMSO	15.2 ± 3.3	12
	LPS, BzATP, S24795 50 µM	9.0 ± 1.4	6
	LPS, BzATP, PNU-282987 10 µM	8.5 ± 1.4	6
	LPS, BzATP, ACh 10 µM	10.0 ± 2.6	6
	LPS, BzATP, PC 200 µM	7.3 ± 2.4	6

Cell death was estimated via measurement of the release of lactate dehydrogenase (LDH) into the cell culture medium. The data depicted in this table correspond to the experiments shown in the respective **Figure 2** of the main part of this manuscript. Monocytic THP-1 cells were primed with lipopolysaccharide (LPS, 1 µg/ml, for 5 h) and further stimulated with 2´(3´)-O-(4-benzoylbenzoyl)adenosine 5´-triphosphate triethylammonium salt (BzATP; 100 µM); The concentration of diverse compounds is indicated in the table. ACh, acetylcholine; DMSO, dimethyl sulfoxide; PC, phosphocholine; SD, standard deviation.

Supplementary Table S2: Cell death in monocytic THP-1 cells as estimated by the lactate dehydrogenase (LDH) activity in cell culture supernatants.

Treatment	Cell death [%] mean ± SD	n
-	4.6 ± 0.9	5
LPS	3.0 ± 0.7	5
LPS, nigericin 50 µM ± DMSO	12.9 ± 2.3	10
LPS, nigericin 50 µM, S24795 50 µM	10.8 ± 1.9	5
LPS, nigericin 50 µM, PNU-282987 10 µM	13.8 ± 2.5	5
Nigericin 50 µM	8.0 ± 4.8	5

Cell death was estimated via measurement of the release of lactate dehydrogenase (LDH) into the cell culture medium. The data depicted in this table correspond to the experiments shown in **Supplementary Figure S2**. Monocytic THP-1 cells were primed with lipopolysaccharide (LPS, 1 µg/ml, for 5 h) and further stimulated with nigericin (50 µM). The concentration of diverse compounds is indicated in the table. DMSO, dimethyl sulfoxide; SD, standard deviation.

Supplementary Table S3: Cell death in monocytic THP-1 cells and THP-1 cell-derived M1-like macrophages as estimated by the lactate dehydrogenase (LDH) activity in cell culture supernatants.

THP-1 cells	Treatment	Cell death [%] mean ± SD	n
**monocytic**	-	5.2 ± 2.6	6
	LPS	5.3 ± 2.5	6
	LPS, BzATP ± DMSO	3.7 ± 2.3	12
	LPS, BzATP, S24795 50 µM	4.0 ± 2.3	6
	LPS, BzATP, S24795 50 µM, MLA 100 µM	3.5 ± 2.1	6
	LPS, BzATP, S24795 50 µM, MLA 50 µM	4.0 ± 2.8	6
	LPS, BzATP, S24795 50 µM, MAL 10 µM	4.2 ± 2.5	6
	LPS, BzATP, ACh 10 µM	4.2 ± 2.7	6
	LPS, BzATP, ACh 10 µM, MLA 100 µM	2.5 ± 1.4	6
	LPS, BzATP, ACh 10 µM, MLA 50 µM	4.0 ± 2.4	6
	LPS, BzATP, ACh 10 µM, MLA 10 µM	4.5 ± 3.5	6
	LPS, BzATP, PC 200 µM	4.0 ± 2.7	6
	LPS, BzATP, PC 200 µM, MLA 100 µM	3.0 ± 1.9	6
	LPS, BzATP, PC 200 µM, MLA 50 µM	4.2 ± 2.5	6
	LPS, BzATP, PC 200 µM, MLA 10 µM	3.7 ± 2.0	6
	LPS, BzATP, MLA 100 µM	4.2 ± 2.9	6
	LPS, BzATP, MLA 50 µM	3.8 ± 2.8	6
	LPS, BzATP, MLA 10 µM	4.2 ± 2.4	6
**M1-like**	-	9.0 ± 2.5	6
	LPS	8.5 ± 2.7	6
	LPS, BzATP ± DMSO	22.9 ± 4.6	12
	LPS, BzATP, S24795 50 µM	23.2 ± 4.3	6
	LPS, BzATP, S24795 50 µM, MLA 50 µM	22.2 ± 6.1	6
	LPS, BzATP, PNU-282987 10 µM	32.2 ± 8.0	6
	LPS, BzATP, PNU-282987 10 µM, MLA 50 µM	33.7 ± 10.3	6
	LPS, BzATP, ACh 10 µM	20.7 ± 3.3	6
	LPS, BzATP, ACh 10 µM, MLA 50 µM	22.7 ± 5.0	6
	LPS, BzATP, PC 200 µM	20.7 ± 3.7	6
	LPS, BzATP, PC 200 µM, MLA 50 µM	25.2 ± 3.7	6
	LPS, BzATP, MLA 50 µM	23.0 ± 5.6	6
	LPS, S24795 50 µM	15.2 ± 4.5	6
	LPS, PNU-282987 10 µM	7.7 ± 1.0	6
	LPS, ACh 10 µM	7.7 ± 0.5	6
	LPS, PC 200 µM	8.0 ± 0.9	6
	LPS, MLA 50 µM	8.8 ± 1.7	6

Cell death was estimated via measurement of the release of lactate dehydrogenase (LDH) into the cell culture medium. The data depicted in this table correspond to the experiments shown in the respective **Figure 3** of the main part of this manuscript. Monocytic THP-1 cells were primed with lipopolysaccharide (LPS, 1 µg/ml, for 5 h) and further stimulated with 2´(3´)-O-(4-

benzoylbenzoyl)adenosine 5´-triphosphate triethylammonium salt (BzATP; 100 µM); The concentration of diverse compounds is indicated in the table. ACh, acetylcholine; DMSO, dimethyl sulfoxide; MLA, methyllycaconitine; PC, phosphocholine; SD, standard deviation.

Supplementary Table S4: Cell death in monocytic THP-1 cells and THP-1 cell-derived M1-like macrophages as estimated by the lactate dehydrogenase (LDH) activity in cell culture supernatants.

THP-1 cells	Treatment	Cell death [%] mean ± SD	n
**monocytic**	-	5.0 ± 0.9	9
	LPS	5.2 ± 1.6	9
	LPS, BzATP ± DMSO	4.0 ± 1.3	18
	LPS, BzATP, S24795 50 µM	4.1 ± 1.0	9
	LPS, BzATP, S24795 50 µM, ArIB 500 nM	4.7 ± 0.8	9
	LPS, BzATP, S24795 50 µM, RgIA4 200 nM	4.2 ± 1.0	9
	LPS, BzATP, PNU-282987 10 µM	5.1 ± 1.3	9
	LPS, BzATP, PNU-282987 10 µM, ArIB 500 nM	4.2 ± 1.0	9
	LPS, BzATP, PNU-282987 10 µM, RgIA4 200 nM	5.7 ± 1.9	9
**M1-like**	-	9.7 ± 1.5	6
	LPS	9.0 ± 1.8	6
	LPS, BzATP ± DMSO	24.3 ± 4.8	12
	LPS, BzATP, S24795 50 µM	12.6 ± 4.3	6
	LPS, BzATP, S24795 50 µM, ArIB 500 nM	25.5 ± 5.3	6
	LPS, BzATP, S24795 50 µM, RgIA4 200 nM	23.8 ± 4.8	6
	LPS, BzATP, PNU-282987 10 µM	16.8 ± 6.7	6
	LPS, BzATP, PNU-282987 10 µM, ArIB 500 nM	21.8 ± 5.1	6
	LPS, BzATP, PNU-282987 10 µM, RgIA4 200 nM	22.8 ± 5.3	6

Cell death was estimated via measurement of the release of lactate dehydrogenase (LDH) into the cell culture medium. The data depicted in this table correspond to the experiments shown in the respective **Figure 4** of the main part of this manuscript. Monocytic THP-1 cells were primed with lipopolysaccharide (LPS, 1 µg/ml, for 5 h) and further stimulated with 2´(3´)-O-(4-benzoylbenzoyl)adenosine 5´-triphosphate triethylammonium salt (BzATP; 100 µM); The concentration of diverse compounds is indicated in the table. ArIB, ArIB[V11L,V16D]; DMSO, dimethyl sulfoxide; SD, standard deviation.
